# GABPA-activated TGFBR2 transcription inhibits aggressiveness but is epigenetically erased by oncometabolites in renal cell carcinoma

**DOI:** 10.1186/s13046-022-02382-6

**Published:** 2022-05-12

**Authors:** Zhiqing Fang, Ning Zhang, Xiaotian Yuan, Xiangling Xing, Xiaofeng Li, Xin Qin, Zhengfang Liu, Shiyong Neo, Cheng Liu, Feng Kong, Magnus Björkholm, Yidong Fan, Dawei Xu

**Affiliations:** 1 Department of Urology, Qilu Hospital of Shandong University, Jinan, China; 2 Department of Medicine, Division of Hematology, Bioclinicum and Center for Molecular Medicine, Karolinska Institute and Karolinska University Hospital Solna, Stockholm, Sweden; 3 Department of Breast Surgery, General Surgery, Qilu Hospital of Shandong University, Jinan, Shandong China; 4 Laboratory Animal Center, Shandong Provincial Hospital Affiliated to Shandong First Medical University, Jinan, China; 5 Department of Oncology-Pathology, Karolinska Institutet, Stockholm, Sweden; 6 Department of Urology, the Third Hospital of Peking University, Beijing, People’s Republic of China; 7 Renal Regeneration Laboratory, Shandong Provincial Hospital Affiliated to Shandong First Medical University, Jinan, China

**Keywords:** ccRCC, GABPA, L-2-HG, Oncometabolite, TGFBR2

## Abstract

**Background:**

The ETS transcription factor GABPA has long been thought of as an oncogenic factor and recently suggested as a target for cancer therapy due to its critical effect on telomerase activation, but the role of GABPA in clear cell renal cell carcinoma (ccRCC) is unclear. In addition, ccRCC is characterized by metabolic reprograming with aberrant accumulation of L-2-hydroxyglurate (L-2HG), an oncometabolite that has been shown to promote ccRCC development and progression by inducing DNA methylation, however, its downstream effectors remain poorly defined.

**Methods:**

siRNAs and expression vectors were used to manipulate the expression of GABPA and other factors and to determine cellular/molecular and phenotypic alterations. RNA sequencing and ChIP assays were performed to identify GABPA target genes. A human ccRCC xenograft model in mice was used to evaluate the effect of GABPA overexpression on in vivo tumorigenesis and metastasis. ccRCC cells were incubated with L-2-HG to analyze GABPA expression and methylation. We carried out immunohistochemistry on patient specimens and TCGA dataset analyses to assess the effect of GABPA on ccRCC survival.

**Results:**

GABPA depletion, although inhibiting telomerase expression, robustly enhanced proliferation, invasion and stemness of ccRCC cells, whereas GABPA overexpression exhibited opposite effects, strongly inhibiting in vivo metastasis and carcinogenesis. TGFBR2 was identified as the GABPA target gene through which GABPA governed the TGFβ signaling to dictate ccRCC phenotypes. GABPA and TGFBR2 phenocopies each other in ccRCC cells. Higher GABPA or TGFBR2 expression predicted longer survival in patients with ccRCC. Incubation of ccRCC cells with L-2-HG mimics GABPA-knockdown-mediated phenotypic alterations. L-2-HG silenced the expression of GABPA in ccRCC cells by increasing its methylation.

**Conclusions:**

GABPA acts as a tumor suppressor by stimulating TGFBR2 expression and TGFβ signaling, while L-2-HG epigenetically inhibits GABPA expression, disrupting the GABPA-TGFβ loop to drive ccRCC aggressiveness. These results exemplify how oncometabolites erase tumor suppressive function for cancer development/progression. Restoring GABPA expression using DNA methylation inhibitors or other approaches, rather than targeting it, may be a novel strategy for ccRCC therapy.

**Supplementary Information:**

The online version contains supplementary material available at 10.1186/s13046-022-02382-6.

## Background

Renal cell carcinoma (RCC) is diagnosed in up to 300 000 people worldwide each year, with clear cell RCC (ccRCC) as the predominant histological subtype (∼80% of all RCCs) [1–3]. ccRCC originates from the epithelial cells of the proximal convoluted tubule in the nephron and the inactivation of the *von Hippel Lindau (VHL)* gene is the early event to drive the disease pathogenesis [4, 5]. VHL serves in the E3 ubiquitin ligase complex, which mediates α subunits of hypoxia-inducible factors (HIF1α and 2α) for proteasomal degradation, while the VHL inactivation leads to the stabilization of HIFαs even under normoxia, thereby resulting in pseudohypoxic phenotype and metabolic reprogramming, including aberrant glycolysis, nucleotide and lipid biosynthesis [5, 6]. In addition, alterations also occur frequently in other genes encoding metabolic enzymes [6]. All these aberrations together give rise to the abnormal increase of certain metabolites with oncogenic function (so-called oncometabolites). For instance, the loss of the *L-2-hydroxyglutarate dehydrogenase (L2HGDH)* gene, and gain of the *malate dehydrogenases (MDHs)* or *lactate dehydrogenases (LDHs)* genes result in the accumulation of L-2-hydroxyglurate (L-2-HG), a *bona fide* oncometabolite that inhibits a group of enzymes of α-ketoglutarate-dependent dioxygenases including the ten-eleven translocation (TET) family of 5-methylcytosine (5mC) hydroxylases, and histone lysine or RNA demethylases [6–10]. The L-2-HG-mediated inhibition of TET activity triggers widespread DNA cytosine hypermethylation in ccRCC, thereby promoting aggressive disease [6, 7, 11, 12]. However, it remains poorly defined how such aberrant DNA hypermethylation contributes to ccRCC progression and which genes are targeted by L-2-HG.

GA-binding protein A (GABPA) is the ETS family transcription factor, and it forms a complex with its partner either GABPB1 or GABPB2 to regulate target gene transcription [13, 14]. GABPA has long been shown to play oncogenic roles in the pathogenesis of leukemia, prostate cancer, glioblastoma and other malignancies [13, 15–17]. More recently, GABPA was further identified as a key transcription factor to activate the mutated telomerase reverse transcriptase (TERT) promoter [18]. TERT promoter mutations occur in various types of cancer and create de novo ETS binding motifs recognized by the GABPA-containing complex [18]. The interplay between the mutated TERT promoter and GABPA induces the expression of TERT, a rate-limiting component for telomerase activity required for immortalization and malignant transformation [14, 18–20]. In glioblastoma cells harboring the TERT promoter mutation, GABPB1 depletion led to reduced TERT expression coupled with diminished telomerase activity followed by progressive telomere attrition and eventual loss of oncogenic potential [17]. Given all the above findings, GABPA or its partner GABPB1 has been suggested as a therapeutic target for tumors bearing TERT promoter mutations [17].

TERT promoter mutations occur in up to 20% of RCCs [4, 21], and it is currently unclear which effects GABPA exerts on ccRCC. The present study is designed to address this issue by combining the ccRCC-specific metabolic reprogramming. By doing so, we observe that GABPA acts as a tumor suppressor by stimulating TGFBR2 transcription and TGFβ signaling, while the oncometabolite L-2-HG epigenetically inhibits GABPA expression, disrupting the GABPA-TGFβ loop to drive ccRCC aggressiveness. Clinically, higher GABPA is significantly associated with longer survival of ccRCC patients. These findings may have important implications in understanding ccRCC pathogenesis and precision oncology.

## Methods

### Patients and specimens

The present study included 31 patients with ccRCC whose tumors and noncancerous adjacent renal tissues (NTs) were available. NT samples were examined to exclude cancer cell contamination or local metastasis. Specimens were freshly frozen at -80 °C until use. Clinical information for this cohort of patients was summarized in Table S1. In addition, the tissue microarray (TMA), containing 90 ccRCC tissues together with patient clinical characteristics and follow-up data on OS (endpoints: dead or alive) (Table S2), was obtained from Shanghai Outdo Biotech (Shanghai, China). The study was approved by Shandong University Qilu Hospital Ethics Committee.

### The Cancer Genome Atlas (TCGA), GEO and EMBL-EBI cohorts of ccRCC and GEO ChIP-sequencing

The data for the TCGA cohort of ccRCC were downloaded via https://portal.gdc.cancer.gov in Jan. 2021 [22]. Patient clinical characteristics were presented in Table S3. mRNA abundances were expressed as RSEM (RNA-Seq by Expectation Maximization). DNA methylation was expressed as β values (the ratio of signal intensity between methylated and unmethylated CpGs). GSEA and KEGG analyses were performed using the ‘clusterProfiler’ R package.

RNA expression data in two other cohorts of ccRCC tumors were downloaded from GEO (https://www.ncbi.nlm.nih.gov/geo/query/acc.cgi?acc=GSE73731) and EMBL-EBI databases (https://www.ebi.ac.uk/arrayexpress/experiments/E-MTAB-1980) in April, 2022. GSE73731 dataset contained 265 ccRCC tumors analyzed using the Affymetrix microarrays [23], while RNA sequencing was performed on 101 ccRCC tumors in E-MTAB-1980 [24].

GSE96015 and GSE105431 data were downloaded from GEO datasets, which contain the GABPA ChIP-seq results obtained from HepG2 cells (GSM2527661 and GSM2527662) and K562 cells (GSM2825959 and GSM2825960), respectively [25].

### Cell lines and cell culture

ccRCC-derived cell lines A498 (purchased from American Type Culture Collection, Manassas, VA) and 786-O cells (Shanghai Institute of Biochemistry and Cell Biology Cell Bank/Stem Cell Bank, Shanghai, China) were used in the present study. Cells were cultured in RPMI-1640 medium (Thermo Fisher Scientific, Waltham, MA) supplemented with 10% FBS (Thermo Fisher Scientific), 100 U/ml penicillin, 100 μg/ml streptomycin and 4 mM L-glutamine. Cells were analyzed for mycoplasma infection every six months.

### Pyrosequencing for DNA methylation analyses

Genomic DNA was extracted using QIAamp DNA blood Mini Kit (Qiagen, Hilden, Germany) and then converted by Sodium Bisulfite using EpiTect Bisulfite Kits (Qiagen, Hilden, Germany). PCR amplification was performed with GABPA methylation region-specific primers. The PCR product was purified by binding to streptavidin-coated Sepharose beads (GE Healthcare, UK), denaturation and washing. Then the sequencing primer was annealed to the purified PCR fragment followed by pyrosequencing in a PyroMark Q96 (Qiagen). The primer sequences are listed in Table S4.

### 5-hmC assays

Quest 5-hmC DNA ELISA kits (Zymo Research) were used to assess 5-hmc levels according to the manufacturer’s protocol. Anti–5-hydroxymethylcytosine polyclonal antibody was pre-coated on the bottom of wells, and 100 ng genomic DNA was denatured and then added. Anti-DNA HRP antibody and HRP developer were employed to detect DNA bound to the anti–5-hmC Ab. Greenish-blue color was analyzed in the wells by a plate reader at 405- to 450-nm detection. The levels of 5-hmC were expressed as absorbance.

### Cell proliferation analyses

Proliferation of ccRCC-derived cells was monitored and analyzed every 8 h for a total of 72 h using IncuCyte S3 Live-Cell Analysis System (Essen Bioscience, Ann Arbor, MI, USA). The changes of phase area confluence represent the cell proliferation.

### Flow cytometry

For cell cycle analyses, cells were fixed with 70% ethanol at + 4 °C overnight and stained with RNAse A (0.5 μg)-containing Propidium Iodide (50 μg/ml). Cell cycle distribution was determined using flow cytometry with ModFit (BD Biosciences, Franklin Lakes, NJ). The assessment of ccRCC stem cell markers CD44 and CD105 was performed by staining cells with FITC-conjugated anti-CD44 (BioLegend) and anti-CD105 (BD Biosciences) antibodies, and fluorescence signals were measured as the expression level of CD44 and CD105, respectively. CD90 expressed was assessed as above using CD99 antibody (BioLegend).

### Spheroid formation assay

Cells (3000/well) were cultured in ultra-low-attachment 96-well plates (Corning Life Sciences) with 100 μl RPMI-1640/10 mM HEPES serum-free medium supplemented with cocktails of following growth factors: 10 ng/mL bFGF (PeproTech Nordic, Stockholm, Sweden) and 20 ng/mL EGF (PeproTech Nordic). Fresh medium was supplemented every 3 days. Fifteen days later, the spheroid colonies were examined under light microscopy, counted and photographed. Single spheroid of ccRCC-derived cells was monitored and analyzed by using IncuCyte 3D Single Spheroid Assays (Essen Bioscience, Ann Arbor, MI, USA). The single spheroid was examined under the IncuCyte, measured and photographed.

### Wound healing assays

Migration of ccRCC-derived cells was monitored and analyzed every 8 h for a total of 40 h using Incucyte Cell Migration Scratch Wound Analysis (Essen Bioscience, Ann Arbor, MI, USA). The changes of the wound region represent the cell migration.

### Matrigel-coated invasion assays

Fifty μl of matrigel (Corning Life Sciences, Flintshire, UK) was first loaded to the bottom of the upper chamber. The cell suspension containing 5.0 × 10^4^ cells/ml was prepared in the serum-free medium and then seeded into the upper chamber. The low chamber contained RPMI-1640 medium with 20% FBS. Cells were incubated at 37 °C for 48 h. Cells invading through the matrigel were stained with crystal violet, counted under the microscope and photographed.

### RNA extraction, reverse transcription and qPCR

Total RNA was extracted with Trizol-Reagent (Thermo Fisher Scientific), and reversely transcribed using High-Capacity cDNA Reverse Transcription Kit (Thermo Fisher Scientific). qPCR was performed in QuantStudio 7 Flex Real-Time PCR System using SYBR Green (Thermo Fisher Scientific). Levels of target mRNA were calculated based on the ΔCT values and normalized to human β2-M expression. Primers used in this study are documented in Table S4.

### RNA-sequencing

Total RNA was extracted from the GABPA-knockdown and control 786-O cells, and a cDNA library was prepared according to the standard Illumina RNA-seq protocol. The raw data was available from GSE165728. FeatureCounts v1.5.0-p3 was used to count the read numbers mapped to each gene. A fold change > 1.5 and FDR < 0.05 were set as the thresholds for identifying DEGs.

### Western blot analysis

Proteins were extracted using Pierce RIPA Buffer (Thermo Scientific) with 1% Phenylmethanesulfonyl fluoride (Sigma-Aldrich) and quantified with DC Protein Assay (Bio-Rad). Thirty µg of proteins were separated in Mini-PROTEAN TGX Gels (Bio-Rad) and transferred to PVDF membranes using Trans-Blot Turbo Transfer Pack (Bio-Rad). Membranes were blocked with 5% non-fat milk diluted in TBST, and then incubated with primary antibodies and secondary antibodies before imaged with Clarity Max Western ECL Substrate (Bio-Rad, 1,705,062) and ChemiDoc MP Imaging System (Bio-Rad). The following primary antibodies were used: GABPA (Santa Cruz), L2HGDH (Novus), LDHB (Santa Cruz), MDH2 (Santa Cruz), cMYC (Santa Cruz), TGFBR2 (Abcam), SMAD2/3 and p-SMAD2/3 (Sigma-Aldrich), CDKN1A (Cell Signaling Technologies), CDH1 (Cell Signaling Technologies), vimentin (Cell Signaling Technologies) and Actin (Santa Cruz). Secondary antibodies include Goat Anti-Mouse IgG (H + L)-HRP Conjugate and Goat Anti-Rabbit IgG (H + L)-HRP Conjugate (Bio-Rad).

#### Immunofluorescence

Control and GABPA-depleted A498 and 786-O cells were treated with 4% paraformaldehyde followed by 5% BSA. The cells were then incubated with FITC-conjugated GABPA and Alexa Fluor® 594-conjugated TGFBR2 antibodies (Santa Cruz) at room temperature for 2 h, respectively. Cells were finally counterstained with Hoechst and imaged under a fluorescence microscopy.

### Promoter activity assessment

TGFBR2 promoter constructs were made by Shanghai Integrated Biotech Solutions Co. Ltd. Putative GABPA binding sites on the TGFBR2 promoter regions were identified using the Consite software and its mutant variant made using a Site-Directed Mutagenesis Kits (Thermo Fisher Scientific). The above promoter reporters were transfected into A498 and 786-O cells, or co-transfected with *GABPA* expression vectors. Cells were then harvested, and luciferase activity was determined using a dual luciferase reporter assay system (Promega, Madison, USA). The target promoter-driven firefly luciferase activity was normalized to the renilla activity included in the kit.

### Chromatin immunoprecipitation (ChIP)

SimpleChIP® Enzymatic Chromatin IP Kit (Cell Signaling Technologies) was used according to the protocol provided. In brief, cells were crosslinked with formaldehyde. Chromatin digestion was performed with micrococcal nuclease and analyzed by agarose gel. For GABPA binding to the TGFBR2 promoter, antibodies against GABPA (Sigma-Aldrich) were added into the digested samples and incubated overnight at 4 °C with rotation. For 5hmC at the GABPA methylation region spanning cg08521263, antibodies against 5hmC (Abcam) were added. IgG antibodies were included as negative controls in ChIP assays. Protein G magnetic beads were used to precipitate the DNA-antigen–antibody complex followed by the elution of chromatin from Antibody/Protein G magnetic beads and reversal of cross-links. Spin columns were used to purify DNA and the collected DNA was amplified using PCR with specific primers (Table S4).

### Immunohistochemistry (IHC)

Paraffin embedded slides were deparaffinized and rehydrated followed by antigen-retrieval using citric acid buffer. Endogenous peroxidase was deactivated by H_2_O_2_. Slides were blocked using 10% goat serum and incubated with the corresponding primary antibodies overnight at 4 °C. After incubation with secondary antibodies for 45 min at room temperature, DAB staining (Thermo Fisher Scientific) was used to detect the antigen–antibody binding. The primary antibodies used were: GABPA (ProteinTech) and TGFBR2 (Abcam). The slides were examined by two of the co-authors (ZF and NZ) and mean values of GABPA and TGFBR2 positive cells were presented based on the results from two observers. For each slide, a total of 200 cells in two fields were analyzed.

### Nude mice and tumor cell injection

Six-week-old male athymic BALB/c nude mice were purchased from Beijing Vital River Laboratory Animal Technology Co., Ltd./Charles River Laboratories, Beijing, China, and used to evaluate the in vivo effect of GABPA on metastasis and growth. Two millions of 786-O cells expressing ectopic GABPA (786-O/GABPA) and control cells with empty vectors (786-O/Control) were injected into nude mice via the tail vein (4/group) and subcutaneously (10/group), respectively. Mice via vein injection were killed after 8 weeks and lungs were collected for evaluation of tumor seeding or metastasis. Mice with subcutaneous injection were monitored for tumor growth, and when tumors were palpable, their sizes were measured every 4 days for 28 days. Mice were then killed, and tumors were isolated for growth and IHC analyses. The study was approved by Shandong University Qilu Hospital Ethics Committee.

### Statistical analyses

All statistical analyses were performed using IBM SPSS Statistics version 24 (IBM, Armonk, NY). Based on the distribution of data, Student’s t-test, Mann–Whitney U-test, Kruskal Wallis test and Chi^2^-test or Fisher’s exact test were used for analysis. Spearman’s Rank-Order Correlation coefficient was applied to determine correlation coefficients R. Survival analyses were performed with log-rank test. Overall survival (OS) disease-free survival (DFS) was visualized with Kaplan–Meier plots. Univariate and multivariate Cox regression analyses were used to determine the effect of various predictor variables on OS and PFS. *p*-values < 0.05 were considered as statistically significant.

## Results

### Downregulation of GABPA expression occurs in primary ccRCC tumors and is associated with patient survival

**Fig. 1:**
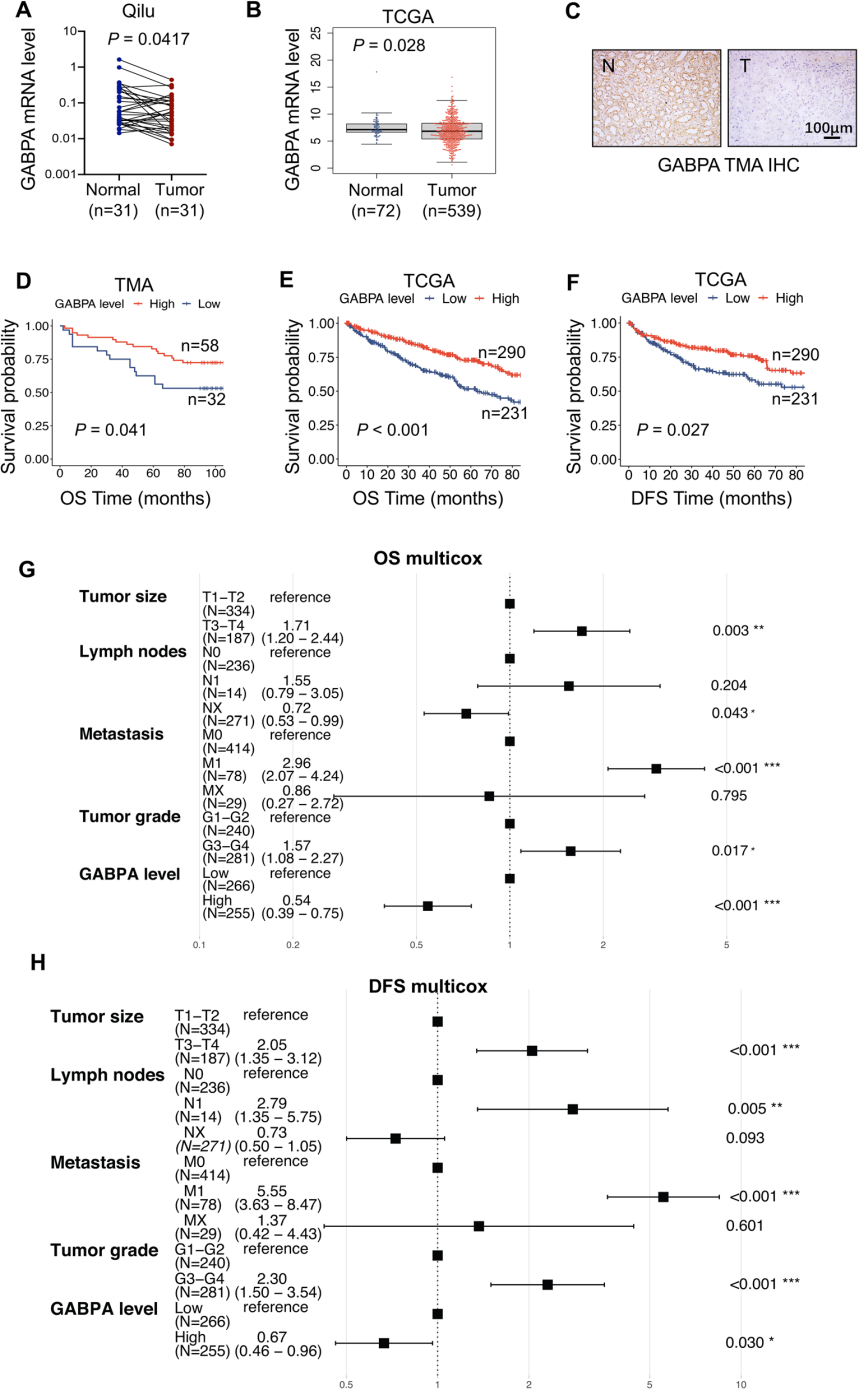
GABPA is downregulated in ccRCC tumors and is associated with patient survival. **A** GABPA expression in tumors and matched noncancerous adjacent renal tissues (NTs) from 31 ccRCC patients. GABPA mRNA expression was determined using qPCR. **B** GABPA mRNA expression in ccRCC tumors and NTs from the TCGA cohort of patients. **C** The representative IHC staining images showed diminished GABPA expression at protein levels in the tumor (T) compared that in matched NT. Scale bar: 100 µm. **D** Higher GABPA expression is associated with longer overall survival (OS) in the TMA cohort of ccRCC patients. **E** and **F** Higher GABPA mRNA expression predicts longer OS and DFS in the TCGA cohort of ccRCC patients. **G** and** H** Multivariate analyses show the impacts of GABPA mRNA expression on OS and DFS in the TCGA cohort of ccRCC patients, respectively

We first compared GABPA expression between ccRCC tumors and matched adjacent non-cancerous renal tissues (NTs). In 31 ccRCC patients whose tumor specimens and NTs were available (Table S1), qPCR results unexpectedly revealed that GABPA mRNA levels were significantly lower in most tumors (22/31) than in NTs (Fig. 1A). Further analyses of GABPA expression in 72 NTs and 539 tumors from the TCGA cohort of ccRCCs showed similar results (NTs vs tumors, GABPA: *P* = 0.028) (Fig. 1B). Consistent with mRNA expression, significantly diminished GABPA protein expression was observed in tumors, as determined using IHC staining (Fig. 1C). Because TERT promoter mutations are frequent in ccRCC, while GABPA is required to activate the mutant promoter [4, 18, 21], we further analyzed TERT expression and observed its upregulation in those ccRCC tumors (NTs vs tumors, *P* < 0.001), which was contrast to downregulation of GABPA in tumors (Fig. S1). GABPA and TERT expression was inversely correlated (*R* = -0.103, *P* = 0.018) (Fig. S1). These findings demonstrate that GABPA expression is markedly downregulated while TERT is robustly induced in ccRCC tumors.

We then evaluated whether GABPA could serve as a prognostic factor for ccRCC. Overall survival (OS) data and tissue array materials were available in 90 ccRCC patients, and their clinical characteristics were summarized in Table S2. The tissue array block was analyzed for GABPA expression, as determined by IHC staining. Patients were categorized into high- and low-GABPA groups (58 and 32 patients, respectively) based on IHC evaluation, and the high-GABPA group patients had a significantly longer OS than did the low-GABPA group (*P* = 0.041) (Fig. 1D). We further assessed the impact of GABPA mRNA expression on OS and disease-free survival (DFS) in the TCGA cohort of ccRCC patients. Clinic-pathological data of patients were listed in Table S3. Univariate analyses revealed that high levels of GABPA expression predicted longer OS and DFS (Fig. E and F). For multivariate analyses that include GABPA expression, tumor size, lymph node (N stage) and distant metastasis, and tumor grade, high-GABPA expression remained as a significant variable associated with longer patient OS [HR = 0.54 (0.39 – 0.75), *P* < 0.001], and DFS [HR = 0.67 (0.46 – 0.96), *P* = 0.03] (Fig. 1G and H).

### GABPA regulates proliferation, stemness and migration/invasion of ccRCC cells

**Fig. 2:**
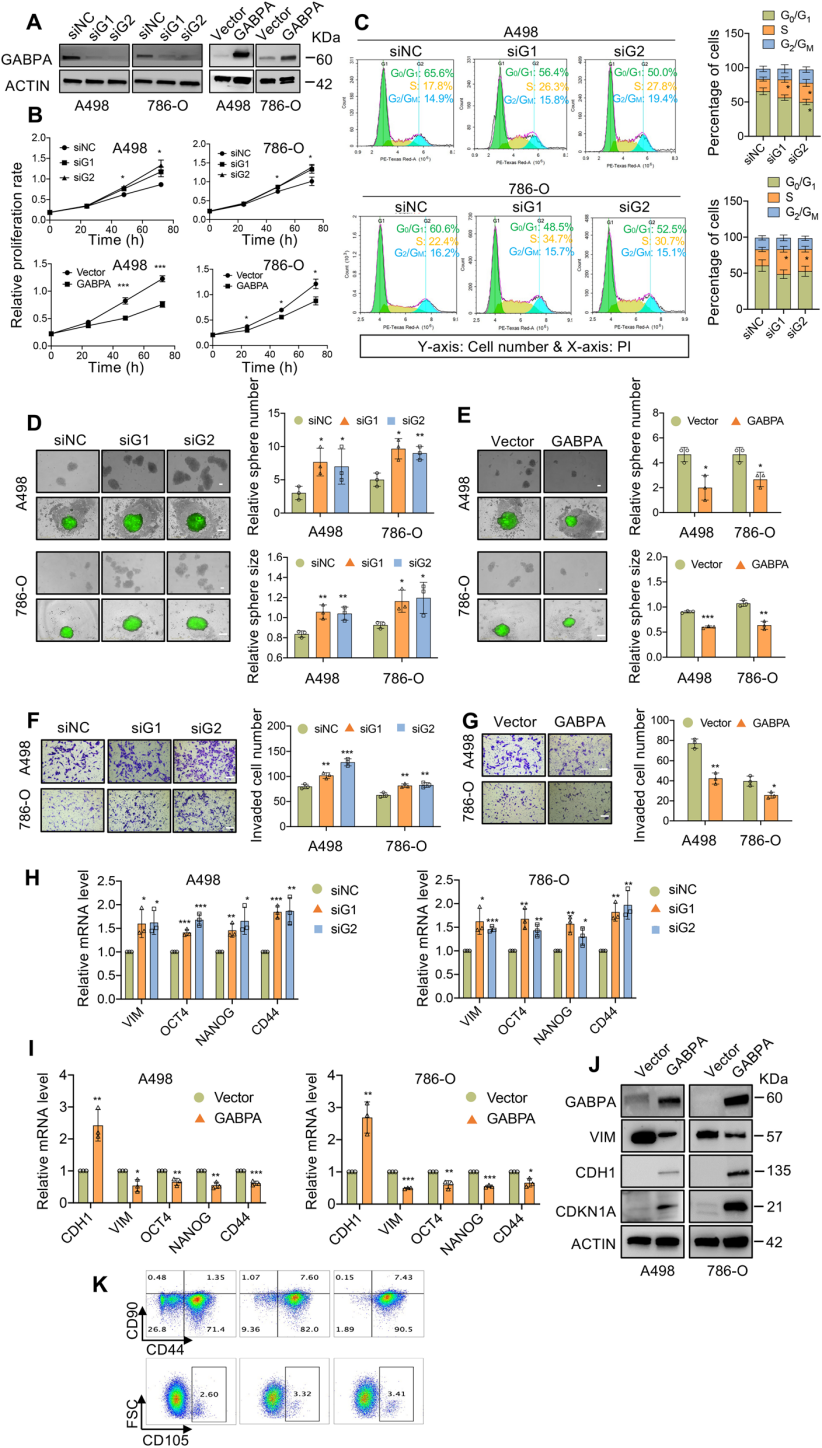
GABPA regulates proliferation, stemness and invasion of ccRCC cells. **A** GABPA-specific siRNAs and its expression vectors efficiently inhibit and overexpress GABPA in A498 and 786-O cells, respectively, as determined using immunoblotting. **B** GABPA depletion promotes while its overexpression inhibits cell proliferation. IncuCyte S3 Live-Cell Analysis System was employed to measure cell proliferation rate. **C** GABPA depletion increases while its overexpression reduces cells at S phase, as assessed using flow cytometry. **D** and **E** GABPA depletion increases while its overexpression reduces the stemness of A498 and 786-O cells. Scale bars: 200 µm. **F** and **G** GABPA depletion increases while its overexpression reduces the invasive capacity of A498 and 786-O cells. Scale bars: 100 µm. (H-J) GABPA regulates the expression of proliferation-, stemness- and EMT-related genes. (K) ccRCC stem cell (CD44 and CD105) and mesenchymal stem cell markers (CD90) are upregulated in GABPA-depleted cells. Three independent experiments were performed. *, ** and *** denote *P* < 0.05, 0.01 and 0.001, respectively

We then sought to determine functional activities of GABPA in ccRCC cells. For this purpose, we chose two ccRCC-derived cell lines, A498 cells with a wt TERT promoter and 786-O cells with a mutated one. First, two siRNAs specifically targeting GABPA mRNA were used to inhibit GABPA expression in A498 and 786-O cells (Fig. 2A left). Consistent with published reports, GABPA inhibition led to significant downregulation of TERT expression in these cells (Fig. S2). Despite diminished levels of TERT, however, A498 and 786-O cells depleted of GABPA proliferated significantly faster than did control cells, with almost one-fold increase of cell numbers during a 72 h-culture period (Fig. 2B top panel). Cell cycle analyses revealed that GABPA-knockdown cells at S phase significantly increased, which was coupled with decline in G0/1 cells (Fig. 2C). In contrast, ectopic GABPA expression inhibited cell proliferation compared to control cells (Fig. 2A right panel and 2B, bottom panel).

The effect of GABPA on stemness and migration/invasion of A498 and 786-O cells was further evaluated. Cell stemness was measured as self-renewal capabilities using spheroid formation assay. Substantially increased sphere numbers were observed in GABPA-depleted cells compared to their control counterparts (Fig. 2D). Quantitative analyses revealed that spheres derived from GABPA-depleted cells were much bigger (Fig. 2D). Whereas ectopic GABPA expression led to significantly reduced numbers and sizes of spheres generated from these cells (Fig. 2E).

Wound healing and Transwell assays were employed to assess cellular migration and invasion. GABPA-knocked-down cells had almost completely closed or much narrower wound areas within 16 h, approximately 50% of wound width in control cells (Fig. S3). In addition, GABPA over-expression slowed down the closure of wound areas (Fig. S3). Importantly, GABPA knockdown significantly increased cells invading through the matrigel-coated membrane compared to the control cells (Fig. 2F). In contrast, numbers of invaded cells with ectopic GABPA expression were significantly reduced (Fig. 2G).

Consistent with phenotypic changes mediated by altered GABPA expression, we further observed that GABPA overexpression induced CDKN1A and E-cadherin expression while inhibited vimentin expression in A498 and 786-O cells (Fig. 2H – 2J). These results indicate that GABPA plays a role in epithelial-mesenchymal transition (EMT). ccRCC stem cell markers CD44 and CD105 were further analyzed using flow cytometry. Expression of both CD44 and CD105 increased significantly in GABPA-knockdown A498 cells (Fig. 2K). In addition, CD90, a mesenchymal stem cell marker, was also upregulated in these cells upon GABPA depletion (Fig. 2K).

### GABPA promotes TGFBR2 gene transcription in ccRCC cells

**Fig. 3:**
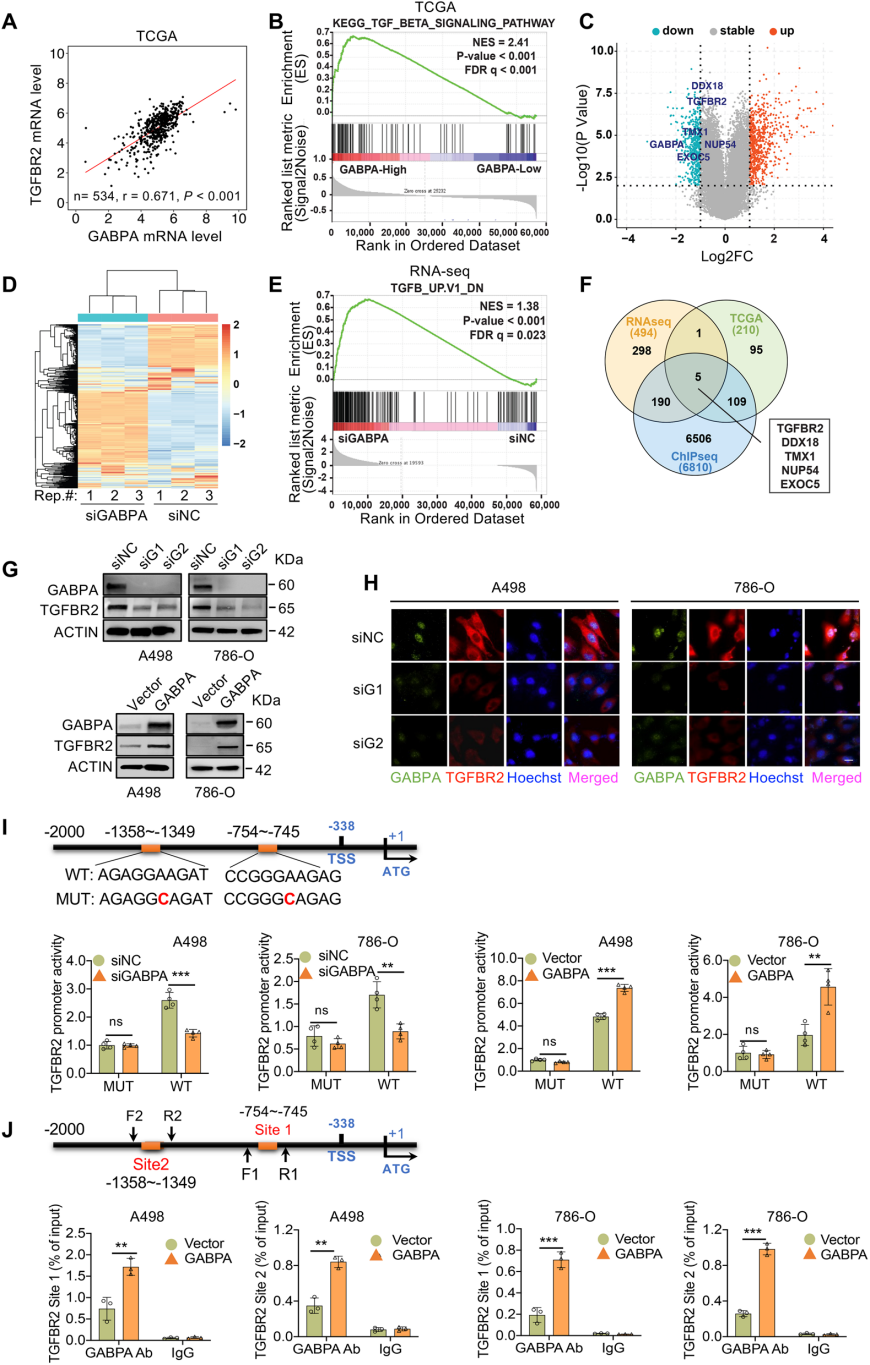
TGFBR2 is the GABPA target gene. **A** GABPA and TGFBR2 expression is highly correlated with each other in the TCGA ccRCC tumors. **B** The GSEA analysis reveals that the TGFβ signaling is enriched in ccRCC tumors expressing higher GABPA in the TCGA cohort of ccRCC. **C** The volcano plot shows TGFBR2 among the top downregulated genes in GABPA-depleted cells, as determined using RNA seq derived from 3 independent experiments (Exp). **D** The heatmap of RNA seq results illustrates significantly downregulated expression of the TGFβ pathway factors. **E** The GSEA analysis of RNA seq results shows the diminished TGFβ pathway enrichment in GABPA-depleted cells. **F** Venn diagram reveals five overlapped genes based on the integrated analyses of TCGA, RNA seq and GABPA ChIP-seq of leukemic and liver cancer cells. **G** GABPA knockdown downregulates while its overexpression upregulates TGFBR2 expression. **H** Immunofluorescence results further reveal diminished TGFBR2 expression in GABPA-depleted cells. Scale bars: 10 µm. **I** Top panel: The schematic drawing shows the GABPA binding motifs on the TGFBR2 promoter and mutated nucleotides. Bottom panel: GABPA knockdown inhibits while its overexpression stimulates the TGFBR2 promoter activity. **J** Top panel: The schematic diagram shows the primer locations spanning GABPA sites for ChIP assay. Bottom panel: The enrichment of GABPA on the TGFBR2 promoter, as determined using ChIP assay. Three independent experiments were performed. *, ** and *** denote *P* < 0.05, 0.01 and 0.001, respectively

Given the findings above, we then sought to decipher how GABPA regulates cellular phenotypes and related molecules in ccRCC. Toward this purpose, we first looked for potential downstream targets of GABPA using in silico approaches: (i) The RNA sequencing or microarray data analyses of TCGA ccRCC tumors. By doing so, we identified TGFBR2 as one of the top genes whose expression was positively correlated with GABPA (Fig. 3A). Moreover, the GSEA analysis of these ccRCC tumors showed that the TGFβ pathway was markedly enriched in tumors expressing higher GABPA (Fig. 3B). In addition, two other cohorts of ccRCC tumors similarly showed a significantly positive correlation between GABPA and TGFBR2 expression (Fig. S4). (ii) RNA sequencing analyses of GABPA-depleted 786-O cells: TGFBR2 was among the top downregulated genes (Fig. 3C), and the TGFβ signal was underrepresented upon GABPA knockdown (Fig. 3D and 3E). (iii) Analyses of available GABPA ChIP-seq data [25]. The ChIP-seq of GABPA-expressing leukemic K562 and liver cancer HepG2 cells identified a total of 6,810 gene promoters bound by GABPA, and five genes were overlapped based on the integrated analyses above, among which was TGFBR2 (Fig. 3F). To corroborate the relationship between GABPA and TGFBR2, we further determined their expression in A498 and 786-O cells. As shown in Fig. 3G and 3H, GABPA depletion and over-expression down- and up-regulated TGFBR2 expression at protein levels, respectively.

To define whether *TGFBR2* is a direct target gene of GABPA, we analyzed the TGFBR2 promoter and identified two consensus binding motifs for GABPA (Fig. 3I). A 2 kb-long TGFBR2 promoter spanning these two motifs was cloned into the pGL3 vector and the vector was then co-transfected with a GABPA expressing plasmid into A498 and 786-O cells. GABPA significantly increased the TGFBR2 promoter activity (Fig. 3I). In contrast, GABPA depletion led to the diminished promoter activity. When the GABPA binding sites were mutated, the TGFBR2 promoter almost completely lost its response to GABPA (Fig. 3I). Moreover, the GABPA binding motif mutation led to dramatic reduction in the basic TFGBR2 promoter activity. We further performed ChIP experiments to determine the GABPA occupancy on the TGFBR2 promoter. As shown in Fig. 3J, GABPA bound to both motifs in the promoter. As described above, the ChIP-seq data derived from GABPA-expressed K562 and HepG2 cells also revealed strong GABPA signals in the TGFBR2 promoter region. Furthermore, the locations of the GABPA peak signals corresponded to where the GGAA motifs (GABPA sites) were present (Fig. S5). Taken together, TGFBR2 is the GABPA target gene.

### The GABPA-TGFBR2 cascade activates the TGFβ signaling to regulate ccRCC cell phenotypes

**Fig. 4:**
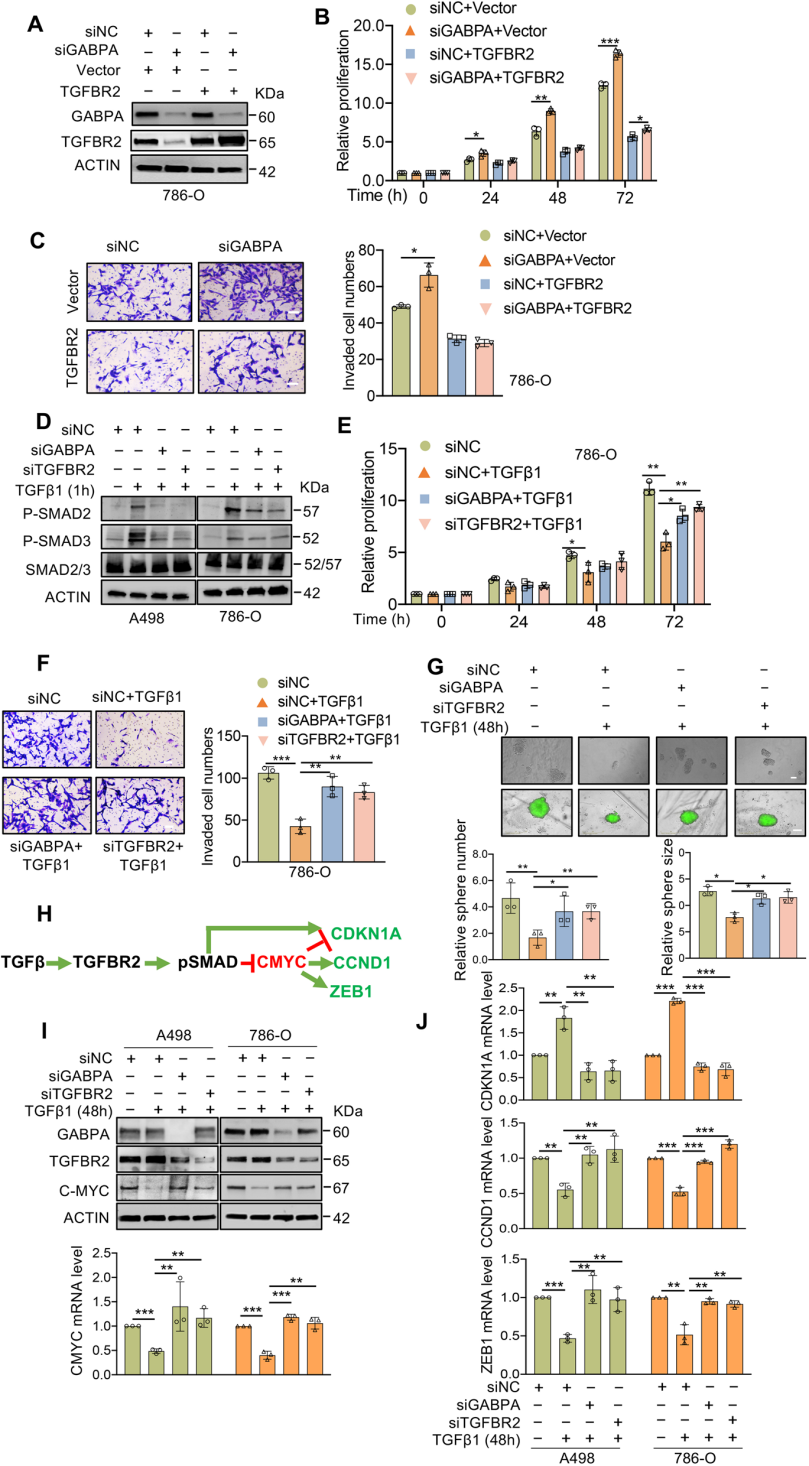
GABPA activates the TGFβ pathway to exert its effects on ccRCC cells. **A** and **B** The accelerated cell proliferation resulting from GABPA knockdown is abolished by TGFBR2 overexpression. **C** The enhanced cell invasion resulting from GABPA knockdown is abolished by TGFBR2 overexpression. Scale bars: 100 µm. **D** TGFβ-mediated SMAD2/3 phosphorylation is abolished by knockdown of either GABPA or TGFBR2. **E** The inhibition of cell proliferation mediated by TGFβ is significantly attenuated by knockdown of GABPA or TGFBR2. **F** The inhibition of cell invasion mediated by TGFβ is significantly attenuated by knockdown of either GABPA or TGFBR2. Scale bars: 100 µm. **G** The stemness or self-renewal inhibition mediated by TGFβ is abolished by knockdown of either GABPA or TGFBR2. Scale bars: 200 µm. **H** The schematic illustration for TGFβ and cMYC targets. **I** TGFβ-mediated downregulation of cMYC is abolished by knockdown of either GABPA or TGFBR2. **J** The expression of TGFβ and/or cMYC target genes are altered in response to TGFβ treatment and knockdown of GABPA or TGFBR2. Three independent experiments were performed. *, ** and *** denote *P* < 0.05, 0.01 and 0.001, respectively

We then explored a functional link between GABPA and TGFBR2. First, we wanted to determine whether TGFBR2 was able to reverse phenotypic alterations mediated by GABPA inhibition. Toward this end, 786-O cells were transfected with TGFBR2 expression vectors followed by GABPA knockdown (Fig. 4A). Ectopic TGFBR2 expression completely erased GABPA depletion-mediated proliferation and invasion acceleration of 786-O cells, and even further lowered proliferation and invasion compared to control cells, likely due to excessive abundance of TGFBR2 (Fig. 4B and 4C).

To further address this issue, we knocked down GABPA and TGFBR2 in A498 and 786-O cells, respectively, and then monitored cellular responses to TGFβ. As expected, TGFβ treatment enhanced SMAD2/3 phosphorylation in control cells, however, this event was attenuated or even abolished in either GABPA- or TGFBR2-depleted cells (Fig. 4D). In the presence of TGFβ, control 786-O cells displayed substantial declines in proliferation, stemness and invasion, while GABPA or TGFBR2 knockdown counteracted all these inhibitory effects of TGFβ (Fig. 4E-4G). Collectively, GABPA and TGFBR2 depletion phenocopies each other through losing a response to TGFβ.

It is well defined that TGFβ transcriptionally promotes expression of CDKN1A while inhibits expression of the oncogene c-MYC (Fig. 4H), and moreover, MYC has many downstream targets including CCND1 and ZEB1 [26, 27]. All these factors are critical regulators of proliferation, stemness and invasion or EMT, and we thus determined their expression in cells above. Incubation of control cells with TGFβ led to notable c-MYC downregulation (Fig. 4I), but in GABPA or TGFBR2 knockdown cells treated with TGFβ, there was no significant alterations in c-MYC levels (Fig. 4I). Similar expression alterations were observed for CCND1 and ZEB1 (Fig. 4J). On the other hand, CDKN1A expression increased significantly in TGFβ-treated control cells, which was abolished by GABPA or TGFBR2 depletion (Fig. 4J). These molecular alterations were highly consistent with the observed changes of cell phenotypes and EMT markers (Fig. 2).

### Lower TGFBR2 expression is associated with shorter survival in ccRCC patients

**Fig. 5:**
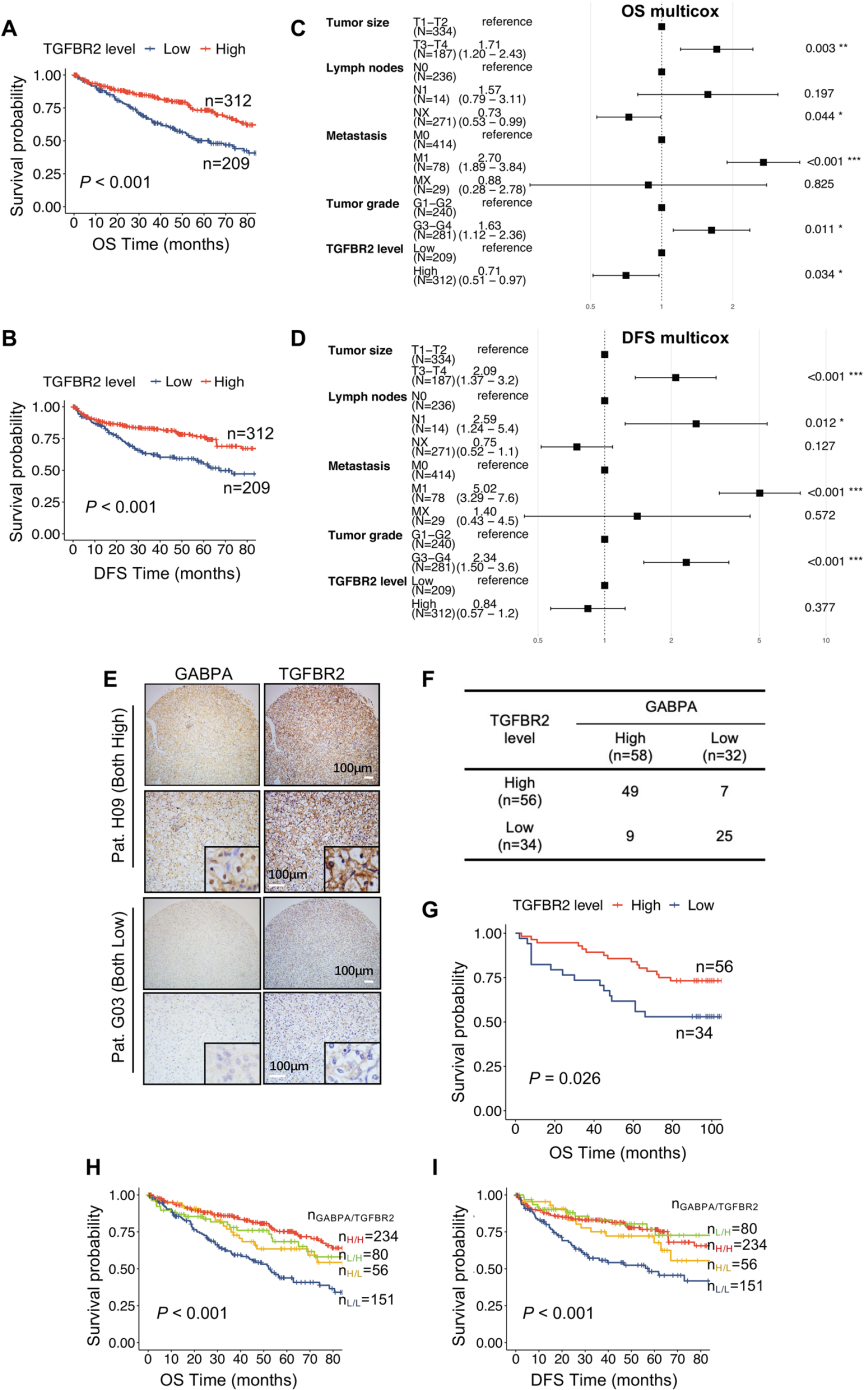
TGFBR2 serves as a prognostic factor for ccRCCs. **A** and **B** Higher TGFBR2 expression predicts longer OS and DFS in the TCGA cohort of ccRCC patients. **C** and **D** Multivariate analyses show the impacts of TGFBR2 on OS and DFS in the TCGA cohort of ccRCC patients, respectively. **E-G** Higher TGFBR2 expression is associated with longer overall survival (OS) in the TMA cohort of ccRCC patients. The representative immunohistochemical results of GABPA-strong and weak tumors and correlation with TGFBR2 (**E** and **F**). Scale bars: 100 µm. (H and I) Patients with both lower GABPA and TGFBR2 had shortest OS and DFS in the TCGA ccRCC cohort

It is currently unclear whether TGFBR2 has a prognostic value in ccRCC. Given the findings above, we hypothesize that TGFBR2, like GABPA, may predict survival in ccRCC. For TCGA cohort of ccRCC patients, higher TGFBR2 mRNA expression was significantly associated longer OS and DFS (Fig. 5A and 5B). Multivariate analyses showed that higher TGFBR2 mRNA expression was still significantly associated with longer patient OS [HR = 0.71 (0.51 – 0.97), *P* = 0.034], but not DFS (Fig. 5C and 5D). Similarly, higher TGFBR2 protein expression predicted longer OS in our cohort of 90 ccRCC patients (*P* = 0.026) (Fig. 5E and 5G). In addition, GABPA and TGFBR2 expression in these tumors, as determined by IHC staining, were highly correlated (Fig. 5F), further supporting their causal relationship. The combination analysis of these two factors showed that the lowest expression of both GABPA and TGFBR2 predicted the shortest OS and DFS in the TCGA ccRCC cohort (Fig. 5H and 5I).

### Downregulation of GABPA expression results from its hypermethylation in ccRCC

**Fig. 6:**
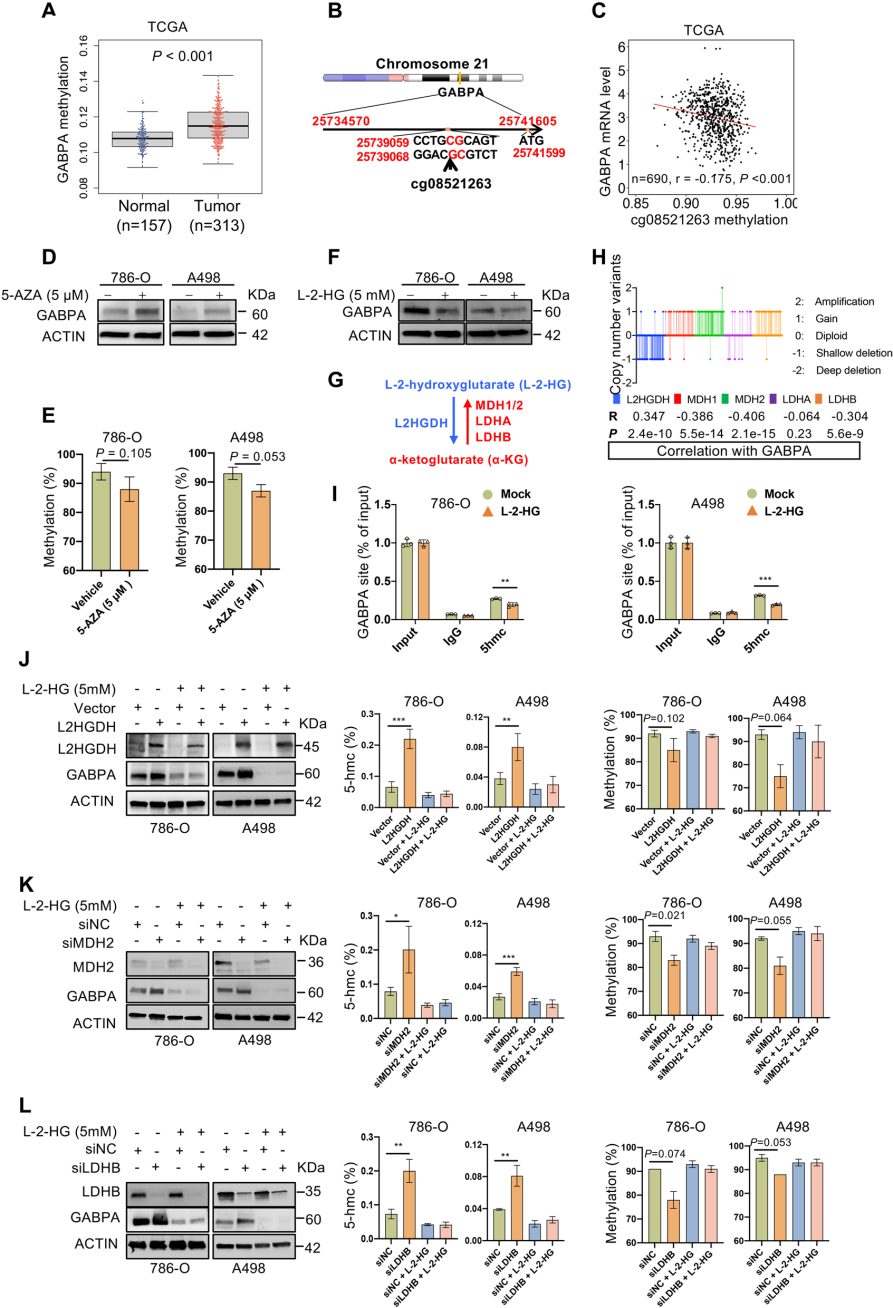
L-2-HG accumulation induces epigenetic silence of GABPA expression. **A** GABPA methylation is significantly higher in ccRCC tumors than in corresponding renal tissues in the TCGA cohort. **B** The schematic illustration of the CpG cg08521263 at the GABPA promoter. **C** The inverse correlation between the methylation of cg08521263 and GABPA mRNA levels in the TCGA cohort. **D** and **E** 5-AZA-treatment of A498 and 786-O cells upregulates GABPA expression coupled with the reduced methylation of cg08521263. Immunoblotting and pyrosequencing were used to assess GABPA expression and methylation, respectively. Three independent experiments were performed. **F** L-2-HG-treatment of A498 and 786-O cells inhibits GABPA expression. **G** The schematic illustration of conversion between L-2-HG and a-KG and related enzymes. **H** The genetic alterations in L2HGDH, MDH1/2, and LDHA/LDHB in tumors and from the TCGA ccRCC cohort and their correlation with GABPA expression. **I** The reduced 5-hmC accumulation in the GABPA sequence spanning cg08521263. Cells were treated with L-2-HG and ChIP assays were then carried out. **J** The restoration of L2HGDH expression upregulates GABPA expression coupled with increased 5-hmc and reduced methylation of cg08521263. All these effects are attenuated by addition of L-2-HG. **K** MDH2 depletion upregulates GABPA expression coupled with increased 5-hmc and reduced methylation of cg08521263. All these effects are attenuated by addition of L-2-HG. **L** LDHB depletion upregulates GABPA expression coupled with increased 5-hmc and reduced methylation of cg08521263. All these effects are attenuated by addition of L-2-HG. Three independent experiments were performed. *, ** and *** denote *P* < 0.05, 0.01 and 0.001, respectively

As ccRCC is a solid tumor characterized by aberrant DNA methylation [11], we then sought to determine whether it is attributable to the observed downregulation of GABPA in ccRCC tumors. To this end, we analyzed the DNA methylation at the *GABPA* loci in the TCGA cohort of ccRCCs and identified significantly increased methylation levels in ccRCC tumors compared with that in NTs (*P* < 0.001) (Fig. 6A). Moreover, one CpG (cg08521263) of GABPA was hypermethylated and inversely correlated with GABPA expression in RCC tumors (*P* < 0.001) (Fig. 6B and 6C). To directly determine a causal relationship between GABPA expression and its methylation, we treated ccRCC-derived A498 and 786-O cells with the DNA methylation inhibitor 5-azacitidine (5-AZA). Indeed, GABPA expression increased robustly in these cells in the presence of 5-AZA (Fig. 6D). Consistent with GABPA upregulation, the methylation at cg08521263 was reduced, although the decline was not statistically significant (at borderline) (Fig. 6E). Likely, the demethylation of other CpGs at the *GABPA* loci also contributes to GABPA upregulation. All these results collectively demonstrate that the GABPA hypermethylation contributes to its downregulation in ccRCCs.

### L-2-HG accumulation mediates GABPA hypermethylation and gene silence in ccRCC

The KEGG analysis of RNA seq data from GABPA-depleted 786-O cells revealed significant enrichments of RCC pathways (Fig. S6), and we thus sought to identify RCC-specific regulators of GABPA. The oncometabolite L-2-HG is known as α-ketoglutarate (α-KG) antagonist competitively inhibiting a-KG/Fe(II)-dependent dioxygenases including the TET family of 5mC hydroxylases, thereby causing aberrant DNA methylation and gene silencing in ccRCC [6, 10, 11]. To determine whether L-2-HG affects GABPA expression, we incubated A498 and 786-O cells with L-2-HG. L-2-HG treatment of these cells led to the downregulation of GABPA (Fig. 6F).

L-2-HG and α-KG are converted with each other by specific enzymes including L2HGDH, MDH1/2 and LDHA/LDHB. L2HGDH catalyzes α-KG generation from L-2-HG while the rest of them do the opposite reaction (Fig. 6G) [6]. To determine the effect of these enzymes on GABPA expression, we first analyzed the expression correlation between them and GABPA in the TCGA ccRCC cohort. GABPA mRNA levels were positively correlated with L2HGDH (*R* = 0.347, *P* = 2.4e-10), while inversely correlated with MDH1, MDH2 and LDHB (*R* = -0.386, -0.406 and -0.304, and *P* = 5.5e-14, 2.1e-15 and 5.6e-9, respectively) (Fig. 6H). There was no correlation between GABPA and LDHA (*P* = 0.23). Therefore, detailed analyses were further performed on L2HGDH, MDH2 and LDHB.

L2HGDH downregulation is widespread due to its genetic deletion in ccRCC [6, 10, 11], and as expected, A498 or 786-O cells express very low levels of L2HGDH (Fig. 6J). We thus transfected these cells with L2HGDH expression vector to restore its expression. The ectopic L2HGDH expression resulted in the upregulation of GABPA expression, coupled with robustly increased 5-hydroxymethylation (5-hmC) levels and reduced cg08521263 methylation, whereas the addition of L-2-HG abolished all these effects of L2HGDH (Fig. 6J). Consistent with these observations, the ChIP assay further demonstrated significantly reduced 5hmC accumulation in the GABPA sequence spanning cg08521263 (Fig. 6I). In contrast, MDH2 and LDHB are frequently overexpressed in ccRCC tumors [6, 10, 11], and therefore, we knocked down the expression of these genes in A498 and 786-O cells using their specific RNAis, respectively. Depletion of each of these two gene products gave rise to the same consequences: substantially enhanced GABPA expression and 5-hmC levels, while reduced cg08521263 methylation (Fig. 6K and 6L). These effects were similarly abolished by the addition of L-2-HG (Fig. 6K and 6L). The results collectively demonstrate that either L2HGDH overexpression and/or inhibition of MDH2 and LDHB contributes to upregulation of GABPA via L-2-HG-mediated alterations in 5-hmC and DNA methylation.

Because L-2-HG, L2HGDH over-expression, and MDH2 or LDHB all regulated GABPA expression, we further evaluated their impacts on ccRCC cell phenotypes. L-2-HG treatment of 786-O cells significantly facilitated proliferation and invasion, which mimicked the effect of GABPA depletion (Fig. S7). In contrast, the restoration of L2HGDH expression and knocking-down of MDH2 or LDKB led to inhibition of cellular proliferation and invasion, while these inhibitory effects were attenuated by addition of L-2-HG (Fig. S7).

### *GABPA over-expression inhibits *in vivo* ccRCC metastasis and carcinogenesis in xenograft mouse models*

**Fig. 7:**
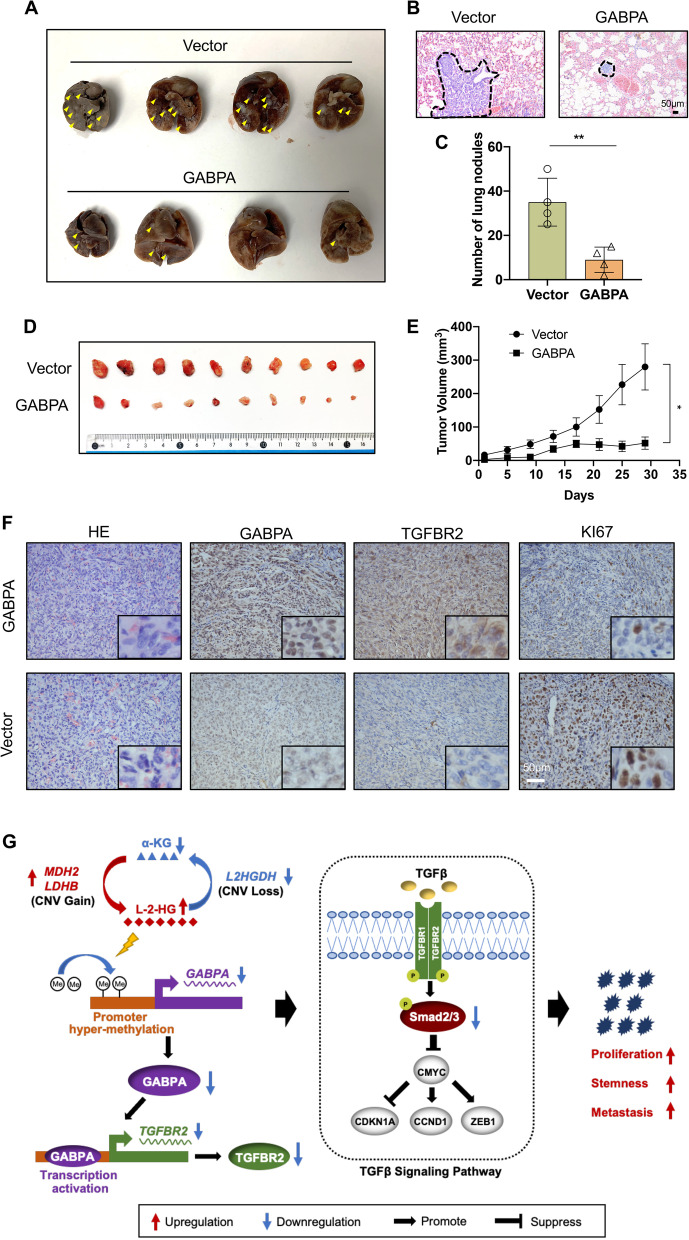
GABPA over-expression inhibits in *vivo* ccRCC metastasis and growth in xenograft mouse models. 786-O/GABPA and 786-O/Control cells were injected into nude mice via the tail vein and subcutaneously, respectively. Lungs and tumors were examined for metastasis and growth, respectively. (**A-C**) Significantly reduced numbers of tumor foci in lungs from mice injected with 786-O/GABPA cells via vein tail. **A** Tumor foci indicated by yellow arrows. **B** H & E staining of metastatic tumors in lungs from 786/control and 786/GABPA cells, respectively. Shown are representative images. Scale bars: 50 µm. **C** The number of tumors in lungs from two groups. **D** and **E** Defective tumor growth in mice subcutaneously injected with 786-O/GABPA cells. **F** IHC analyses of GABPA, TGFBR2 and Ki67 expression in tumors derived from 786-O/control and 786-O/GABPA cells. The images in insets were with bigger magnifications. Scale bars: 50 µm. **G** The work model for the GABPA-TGFβ signaling and relationship with oncometabolites during ccRCC development and progression

To further evaluate the *in viv*o effect of GABPA on ccRCC cells, we conducted tail vein and subcutaneous tumorigenic xenografts in nude mice by injecting 786-O cells transfected with GABPA (786-O/GABPA) and control (786-O/control) vectors, respectively. Lungs and tumors were collected for analyses of tumor cell seeding, growth and IHC staining. The GABPA-overexpressing 786-O cells (786-O/GABPA) gave rise to lung metastasis foci significantly fewer and smaller than those formed in 786-O/control cell-injected mice (Fig. 7A – 7C). Tumors grew significantly slower in mice harboring 786-O/GABPA cells and tumor sizes were only 15% of those derived from 786-O/control cells (Fig. 7D – 7E). The IHC analysis demonstrated higher GABPA and TGFBR2 expression in 786-O/GABPA cell-derived tumors (Fig. 7F). In addition, these tumors expressed substantially lower levels of Ki67, in accordance with their slow growth and weak carcinogenesis (Fig. 7F). Thus, the in vivo metastasis and carcinogenic capacity of 786-O cells were strongly inhibited by GABPA expression.

## Discussion

ccRCC as a metabolic disease exhibits extensive metabolic reprogramming including downregulation of the tricarboxylic acid (TCA) cycle and upregulation of fatty acid synthesis, the pentose phosphate pathway and glutamine transporters, thereby giving rise to aggressive disease and poor prognosis [6, 28]. The aberrant accumulation of L-2-HG, a *bona fide* oncometabolite, has been shown as an important driver for ccRCC progression [6]. Indeed, approximately 1/3 of ccRCC patients already have distant dissemination at presentation, which is in general resistant to conventional chemotherapy and radiotherapy [29]. Although numbers of drugs targeting cellular/molecular pathways have been applied for ccRCC, but patient response to these treatments is limited. Thus, the delineation of ccRCC pathogenesis and identification of new therapeutic targets is demanding tasks. Here we observed a widespread downregulation of GABPA expression in ccRCC and identified TGFBR2 as a direct target of GABPA through which the TGFβ signal was activated to inhibit ccRCC progression. Moreover, GABPA is the downstream effector epigenetically silent by L-2-HG, which disrupted the GABPA-TGFβ loop. Restoring GABPA expression strongly inhibits in vivo metastatic and carcinogenic abilities of ccRCC cells. These results exemplify how oncometabolites rewires a GABPA-mediated signaling and erases its tumor suppressive function for cancer development/progression (Fig. 7G).

TGFBR2 is an established tumor suppressor in several human malignancies [26, 27], however, its role in ccRCC is unclear, although its downregulation has long been observed in ccRCC [30, 31]. The *TGFBR2* gene is localized at chromosome 3p24.1 where LOH is widespread in ccRCC [22]. Intriguingly, the *TGFBR2* LOH does not affect its expression (Fig. S8), indicating that other regulatory mechanisms are more important. In the present study, we provide strong evidence demonstrating TGFBR2 as the direct target of GABPA: (i) GABPA knockdown and over-expression down- and up-regulates TGFBR2 expression, respectively; (ii) GABPA knockdown and over-expression inhibits and stimulates TGFBR2 promoter activity, respectively; moreover, the GABPA binding motif mutation leads to a dramatic reduction in the basic TFGBR2 promoter activity, indicating a key effect of GABPA on TGFBR2 transcription; (iii) The ChIP assay shows the occupancy of GABPA on the TGFBR2 promoter. Similar results were also obtained from the analysis of ChIP-seq databases. TGFBR2 exhibits a tumor suppressive function in ccRCC and attenuates TGFβ response. Consistently, GABPA depletion significantly inhibits the response of ccRCC cells to TGFβ treatment, which includes diminished SMAD2/3 phosphorylation and increased MYC expression, while reduced CDKN1A expression. These TGFβ downstream effectors consequently result in altered proliferation and invasion of ccRCC cells.

2-HG exists as two isoforms including L-2-HG and D-2-HG, and both and α-KG can be converted with each other by specific enzymes [6]. Isocitrate dehydrogenases (IDH1/2) and MDH1/2 or LDHB catalyze α-KG into D-2-HG and L-2-HG, respectively, whereas D2HGDH and L2HGDH oxidize D-2-HG and L-2-HG to α-KG [6]. Gain-of-function of IDH mutations occurs most frequently in low-grade glioma, cartilaginous tumors, intrahepatic cholangiocarcinoma, and certain hematological malignancies; and thus, the accumulation of D-2-HG occurs in these tumors [6]. In contrast, IDH mutations are rare in ccRCC [22]. Instead, loss of L2GHDH or gain of MDHs and LDHB is widespread, which leads to the generation of excess L-2-HG [10, 12, 22]. In addition, pseudohypoxic phenotype-mediated metabolic reprogramming may also contribute to increased L-2-HG [32, 33]. α-KG is a co-substrate for α-KG/Fe(II)-dependent dioxygenases, while L-2-HG acts as an α-KG antagonist to competitively inhibit α-KG/Fe(II)-dependent dioxygenases among which are the TET family of 5mC hydroxylases [6]. The diminished TET activity subsequently results in aberrant DNA methylation in ccRCC [12]. Our findings demonstrate a causal relationship between GABPA hypermethylation/gene silencing and L-2-HG accumulation; the GABPA hypermethylation is coupled with reduced 5hmC, demonstrating impaired TET activity by L-2-HG in ccRCC cells. Of note, ectopic L2HGDH expression or MDH2 and LDHB depletion robustly increased 5hmC, but only inhibited the methylation of cg08521263 moderately. Likely, the demethylation of other CpGs at the *GABPA* loci synergizes with the demethylated cg08521263 to promotes GABPA expression. Indeed, there exist other 14 CpGs at the *GABPA* loci, and 12 of them are largely unmethylated, while the rest two CpGs (cg00106744 and cg21890848) are methylated at low**/**intermediate levels in tumors. Further studies are required to thoroughly elucidate the role of L-2-HG-mediated methylation in inhibiting GABPA expression. In addition, we recognize the global effect of both 5-AZA and L-2-HG on DNA methylation and 5hmC levels, and specific approaches such as dCas9-TET combined with GABPA promoter targeting have been planned to address these issues in our future studies.

Although we identified that the GABPA-TGFBR2 nexus plays a key suppressive role in ccRCC aggressiveness, it remains possible that GABPA may restrain aggressive ccRCC by regulating other targets. For instance, DICER1, a ribonuclease functioning in the microRNA (miRNA) processing machinery, is transcriptionally activated by GABPA through which the invasive phenotype and metastasis of thyroid cancer are inhibited [34]. In bladder cancer, GABPA activates the *Fox1A* and *GATA3* genes, thereby promoting cancer cell differentiation [19]. Thus, further studies are required to thoroughly delineate various effects of GABPA on ccRCC progression. On the other hand, the strong association between GABPA and TGFBR2 is observed in many types of cancer based on the TCGA data analyses, which indicates a broad implication of the present findings in oncogenesis.

Paradoxically, GABPA has long been documented to act as an oncogenic factor, especially for its critical role in activating the mutated TERT promoter and inducing TERT expression [13, 14, 18]. TERT, as the catalytic component of telomerase, is not only required for infinite proliferation of cancer cells by stabilizing telomere length, but also operative in promoting invasion, metastasis and other cancer hallmarks via a telomere lengthening-independent function [19, 20]. Indeed, glioblastoma cells knocked-out of GABPB1, the GABPA partner required for activation of the mutated TERT promoter, was shown to undergo telomere shortening, senescence or apoptosis and eventual loss of tumorigenesis [17]. Therefore, targeting GABPB1 or GABPA for telomerase-based therapy has been suggested as a novel anti-cancer strategy [17, 35]. However, evidence has recently accumulated that GABPA function may be context-dependent, and it acts as a tumor suppressor in several types of cancer, despite its stimulatory effect on TERT transcription [19, 34, 36–39]. Similarly, GABPB1 downregulation due to the gene hypermethylation is observed in thyroid carcinoma and its inhibition promotes invasion of thyroid cancer cells, too [40]. These unprecedented findings suggest that it should be cautious in targeting GABPA or GABPB1 for cancer intervention.

## Conclusions

The present findings demonstrate that GABPA activates TGFBR2 transcription, and thereby enhances the TGFβ signaling to inhibit proliferation, stemness and invasion of ccRCC cells. The inhibitory effect of GABPA expression on in vivo metastatic and carcinogenic capacity is even more robust. The downregulation of GABPA expression due to the gene hypermethylation is widespread in ccRCC tumors, which is attributable to the accumulation of L-2-HG, a *bona fide* oncometabolite. Such lower levels of GABPA expression are associated with poor patient outcomes. Thus, GABPA functions as a tumor suppressor in ccRCC and oncometabolite-mediated epigenetic silencing of GABPA is a critical driver event in the ccRCC progression. These findings not only contribute to better understanding of the ccRCC pathogenesis but are also implicated in ccRCC precision oncology. Based on both in vitro and in vivo results, restoring GABPA expression may be a compelling therapeutic strategy for aggressive ccRCC.

## Supplementary Information

**Additional file 1:TableS1.** Characteristics of 31 ccRCC patients with matched tumor and non-tumoroustissues.

**Additional file 2: Table S2.** Clinic-pathological data of 90 ccRCC patients contained intumor tissue array.

**Additional file 3: Table S3.** Clinic-pathological characteristics of the TCGA cohort of 537ccRCC patients.

**Additional file 4: Table S4.** Sequences of primers, siRNAs and plasmidsused in the study.

**Additional file 5: Figure S1.** The inverse correlation between TERT and GABPAexpression in primary ccRCC tumors from the TCGA cohort.

**Additional file 6: Figure S2.** Downregulation of TERT expression inGABPA-depleted A498 and 786-O cells.

**Additional file 7: Figure S3.** GABPA overexpression inhibits while itsdepletion promotes the migration of ccRCC-derived cells, respectively.

**Additionalfile 8: Figure S4.** The correlationbetween GABPA and TGFBR2 mRNA expression ccRCC tumor from GSE73731 andE-MTAB-1980 cohorts.

**Additionalfile 9: Figure S5.** ChIP-seq shows strong GABPA signals on the TGFBR2promoter in Leukemic K562 and liver cancer HepG2 cells.

**Additional file 10: Figure S6.** KEGG analyses of RNA seq data from GABPA-depleted 786-O cells show theenrichments of RCC pathways.

**Additionalfile 11: Figure S7.** L-2-HG promotes while L-2HGDH over-expression or MDH2and LDHB depletion inhibit the proliferation and invasion of ccRCC-derivedcells.

**Additional file 12: Figure S8.** The lack of correlation between TGFBR2 mRNA expression and its genecopies.

## Data Availability

RNA-sequencing data generated in this study has been deposited to GEO (GSE165728). Any additional information required to reanalyze the data reported in this paper is available from the corresponding authors upon reasonable request.

## Abbreviations

5-AZA: 5-Azacitidine
5mC: 5-Methylcytosine
α-KG: α-Ketoglutarate
ChIP: Chromatin Immunoprecipitation
ccRCC: Clear cell renal cell carcinoma
DFS: Disease-Free Survival
EMT: Epithelial-Mesenchymal Transition
FBS: Fetal Bovine Serum
HIF: Hypoxia-Inducible Factor
L-2-HG: L-2-Hydroxyglurate
L2HGDH: L-2-Hydroxyglutarate Dehydrogenase
LDHB: Lactate Dehydrogenases
MDH: Malate Dehydrogenase
NT: Non-Cancerous Renal Tissues
OS: Overall Survival
RSEM: RNA-Seq by Expectation Maximization
TERT: Telomerase Reverse Transcriptase
TET: Ten-Eleven Translocation
TCGA: The Cancer genome Atlas
VHL: Von Hippel Lindau
