# Supplementary material for: GABPA-activated TGFBR2 transcription inhibits aggressiveness but is epigenetically erased by oncometabolites in renal cell carcinoma
Source: J Exp Clin Cancer Res. 2022 May 12;41:173. doi: 10.1186/s13046-022-02382-6 (PMC9097325; doi:10.1186/s13046-022-02382-6)
Supplement: Supplementary file 1 — Additional file 1:TableS1. Characteristics of 31 ccRCC patients with matched tumor and non-tumoroustissues. [file 13046_2022_2382_MOESM1_ESM.pdf]

Table S1. Characteristics of 31 ccRCC patients with matched tumor and non-tumorous tissues

Variable	N
Age, mean (SEM[a])	59.7 (2.2)
Sex, *n* (%)	
Male	21 (67.7)
Female	10 (32.3)
Grade, *n* (%)	
Low	16 (51.6)
High	14 (45.2)
Unknown	1(3.2)
AJCC stage[b], *n* (%)	
I	17 (54.8)
II	4 (12.9)
III	7 (22.6)
IV	3 (9.7)
T, *n* (%)	
<T2	17 (54.8)
≥T2	14 (45.2)
Lymph node metastasis, *n* (%)	
Positive	1 (3.2)
Negative	14 (45.2)
Unknown	16 (51.6)

[a]SEM, standard error of mean.

[b]Tumor AJCC stages according to the American Joint Committee on Cancer (AJCC) 7th edition.
