# Supplementary material for: GABPA-activated TGFBR2 transcription inhibits aggressiveness but is epigenetically erased by oncometabolites in renal cell carcinoma
Source: J Exp Clin Cancer Res. 2022 May 12;41:173. doi: 10.1186/s13046-022-02382-6 (PMC9097325; doi:10.1186/s13046-022-02382-6)
Supplement: Supplementary file 2 — Additional file 2: Table S2. Clinic-pathological data of 90 ccRCC patients contained intumor tissue array. [file 13046_2022_2382_MOESM2_ESM.pdf]

Table S2. Clinic-pathological data 90 ccRCC patients contained in tumor tissue array

	Micro-Tissue Array	
	Alive<br>(*n* = 59)	Deceased<br>(*n* = 31)
Age, mean (SEM[a])	56.0 (1.4)	65.0 (1.9)
Sex, *n* (%)		
Male	40 (69.0)	19 (61.3)
Female	18 (31.0)	12 (38.7)
Grade, *n* (%)		
Low	51 (86.4)	12 (38.7)
High	8 (13.6)	19 (61.3)
AJCC stage[b], *n* (%)		
I	43 (72.9)	12 (38.7)
II	12 (20.3)	12 (38.7)
III	2 (3.4)	4 (12.9)
IV		2 (6.5)
Unknown	2 (3.4)	1 (3.2)
T, *n* (%)		
<T2	45 (76.3)	12 (38.7)
≥T2	14 (23.7)	19 (61.3)
Lymph node metastasis, *n* (%)		
Positive		2 (6.5)
Negative	57 (96.6)	28 (90.3)
Unknown	2 (3.4)	1 (3.2)
Tumor size (cm), *n* (%)		
< 5	34 (57.6)	9 (29.0)
≥ 5	25 (42.4)	22 (71.0)

[a]SEM, standard error of mean.

[b]Tumor AJCC stages according to the American Joint Committee on Cancer (AJCC) 7th edition.
