# Supplementary material for: GABPA-activated TGFBR2 transcription inhibits aggressiveness but is epigenetically erased by oncometabolites in renal cell carcinoma
Source: J Exp Clin Cancer Res. 2022 May 12;41:173. doi: 10.1186/s13046-022-02382-6 (PMC9097325; doi:10.1186/s13046-022-02382-6)
Supplement: Supplementary file 3 — Additional file 3: Table S3. Clinic-pathological characteristics of the TCGA cohort of 537ccRCC patients. [file 13046_2022_2382_MOESM3_ESM.pdf]

Table S3. Clinic-pathological characteristics of the TCGA cohort of 537 ccRCC patients

	TCGA-KIRC	
	Alive<br>(*n* = 360)	Deceased<br>(*n* = 177)
Age, mean (SEM[a])	58.8 (0.6)	64.2 (0.9)
Sex, *n* (%)		
Male	234 (65.0)	112 (63.3)
Female	126 (35.0)	65 (36.7)
Grade, *n* (%)		
Low	199 (55.3)	45 (25.4)
High	154 (42.8)	131 (74.0)
Unknown	7 (1.9)	1 (0.6)
AJCC stage[b], *n* (%)		
I	224 (62.2)	45 (25.4)
II	44 (12.2)	13 (7.3)
III	74 (20.6)	51 (28.8)
IV	16 (4.4)	67 (37.9)
Unknown	2 (0.6)	1 (0.6)
T, *n* (%)		
<T2	225 (62.5)	50 (28.2)
≥T2	135 (37.5)	127 (71.8)
Lymph node metastasis, *n* (%)		
Positive	5 (1.4)	12 (6.8)
Negative	155 (43.1)	85 (48.0)
Unknown	200 (55.5)	80 (45.2)

[a]SEM, standard error of mean.

[b]Tumor AJCC stages according to the American Joint Committee on Cancer (AJCC) 7th edition.
