# Supplementary material for: GABPA-activated TGFBR2 transcription inhibits aggressiveness but is epigenetically erased by oncometabolites in renal cell carcinoma
Source: J Exp Clin Cancer Res. 2022 May 12;41:173. doi: 10.1186/s13046-022-02382-6 (PMC9097325; doi:10.1186/s13046-022-02382-6)
Supplement: Supplementary file 4 — Additional file 4: Table S4. Sequences of primers, siRNAs and plasmidsused in the study. [file 13046_2022_2382_MOESM4_ESM.pdf]

Table S4. Sequences of primers, siRNAs and plasmids used in the study.

**siRNA**		
GABPA (G1)		5'-GGAGCUGAUAGAAAUUGAGAUUGAU-3'
GABPA (G2)		5'-GCAGAGUGCACAGAAGAAAGCAUUG-3'
TGFBR2 (1)		5'-CUGACUUGUUGCUAGUCAUAUUUCA-3'
TGFBR2 (2)		5'-CUCUGAUGAGUGCAAUGACAACAUC-3'
L-2HGDH (1)		5'-GGUGCAACAGUGAAGUAUCUUCAAA
L-2HGDH (2)		5'-ACAAGAAUGUACUAAUUGCAUUCUU-3'
MDH2 (1)		5'-CUGAAGCGUGACGCUUUCAUCAGTA-3'
MDH2 (2)		5'-GAAGCCAUGAUCUGCGUCAUUGCCA-3'
LDHB (1)		5'-AAGUACAGUCCUGAUUGCAUCAUAA-3'
LDHB (2)		5'-UAUGAAGUCAUCAAGCUAAAAGGAT-3'
**QRT-PCR**		
GABPA	Forward	5’- AAGAACGCCTTGGGATACCCT-3’
	Reverse	5’- GTGAGGTCTATATCGGTCATGCT-3’
TGFBR2	Forward	5'-ctg ccc atc cac tga gac at-3'
	Reverse	5'-ctg cag ttg ctc atg cag gat-3'
TERT	Forward	5’-CGGAAGAGTGTCTGGAGCAA-3’
	Reverse	5’-GGATGAAGCGGAGTCTGGA-3’
L-2HGDH	Forward	5’-caa gaa gca ggt ggc tct gtc t-3’
	Reverse	5’-gca cat gtc aca aca tac tga ca-3’
MDH2	Forward	5’-ctg agc cac atc gag acc aa-3’
	Reverse	5’-ccg gaa taa cta cca cat cac a-3’
LDHB	Forward	5’-gga tct gca gca tgg gag ct -3’
	Reverse	5’-cgg act cct gca gtt acc act-3’
cMYC	Forward	5'-TACCCTCTCAACGACAGCAGCTCGCCCAAGTCCT-3'
	Reverse	5'-TCTTGACATTCTCCTCGGTGTCCGAGGACCT-3'
CCND1	Forward	5′-GCC GAG AAG CTG TGC ATC T-3′
	Reverse	5′-CTC CTC CGC CTC TGG CAT T-3′
CDKN1A	Forward	5'-GCGACTGTGATGCGCTAAT-3'
	Reverse	5'-TAGGGCTTCCTCTTGGAGAA-3'
ZEB1	Forward	5′-AGCAGTGAAAGAGAAGGGAATGC-3′
	Reverse	5′-GGTCCTCTTCAGGTGCCTCAG-3′

CDH1	Forward	5′-TTCCTCCCAATACATCTCCC-3′
	Reverse	5′-TTGATTTTGTAGTCACCCACC-3'
Vimentin	Forward	5'-CTC TTC CAA ACT TTT CCT CCC-3'
	Reverse	5'-AGT TTC GTT GAT AAC CTG TCC-3'
OCT.	Forward	5'-GGG TGG AGG CTG ACA ACA-3'
	Reverse	5'-CGG GCA CTG CAG GAA CAA AT-3'
CD44	Forward	5'-GGG GTC TAC ATC CTC ACA TCC A-3'
	Reverse	5'-CGG GTG CCA TCA CGG TTA ACA-3'
NANOG	Forward	5'-cacggagactgtctctcctct-3'
	Reverse	5'-ctgggtggaagagaacacagt-3’
β2-M	Forward	5’-GAATTGCTATGTGTCTGGGT-3’
	Reverse	5’-CATCTTCAAACCTCCATGATG-3’
**ChIP assay**		
TGFBR2 promoter region 1	Forward	5’-cct gga gac cct ggc aca a-3’
	Reverse	5’-gcc ctc tgg tca gta gat ct-3’
TGFBR2 promoter region 2	Forward	5’-ccc acc act atc act tcg tga-3’
	Reverse	5’-cct gga tag agc gtc cca ttt-3’
GABPA promoter cg08521263	Forward	5'-CAG AGG GTC TTC ATT TCT TCA-3'
	Reverse	5'-GGG TAA GGT CTC CAC TGG GA-3’
**Plasmids**		
pGL3-TGFBR2-Promoter reporters (wt)	Shanghai Integrated Biotech Solutions Co.,Ltd	
pGL3-TGFBR2-Promoter reporters (mt)	Shanghai Integrated Biotech Solutions Co.,Ltd	
GABPA expression plasmid	Shanghai Integrated Biotech Solutions Co.,Ltd	
L-2HGDH expression plasmid	Origene	
TGFBR2 expression plasmid	Addgene	
