# Supplementary material for: GABPA-activated TGFBR2 transcription inhibits aggressiveness but is epigenetically erased by oncometabolites in renal cell carcinoma
Source: J Exp Clin Cancer Res. 2022 May 12;41:173. doi: 10.1186/s13046-022-02382-6 (PMC9097325; doi:10.1186/s13046-022-02382-6)
Supplement: Supplementary file 5 — Additional file 5: Figure S1. The inverse correlation between TERT and GABPAexpression in primary ccRCC tumors from the TCGA cohort. [file 13046_2022_2382_MOESM5_ESM.pdf]

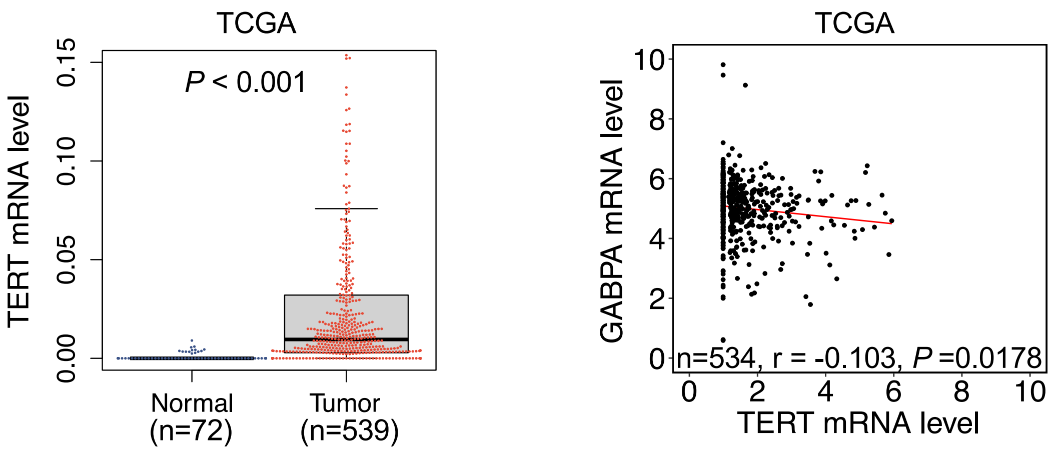

Figure S1. The inverse correlation between TERT and GABPA expression in primary ccRCC tumors from the TCGA cohort. mRNA was expressed as RSEM (RNA-Seq by Expectation Maximization).
