# Supplementary material for: GABPA-activated TGFBR2 transcription inhibits aggressiveness but is epigenetically erased by oncometabolites in renal cell carcinoma
Source: J Exp Clin Cancer Res. 2022 May 12;41:173. doi: 10.1186/s13046-022-02382-6 (PMC9097325; doi:10.1186/s13046-022-02382-6)
Supplement: Supplementary file 6 — Additional file 6: Figure S2. Downregulation of TERT expression inGABPA-depleted A498 and 786-O cells. [file 13046_2022_2382_MOESM6_ESM.pdf]

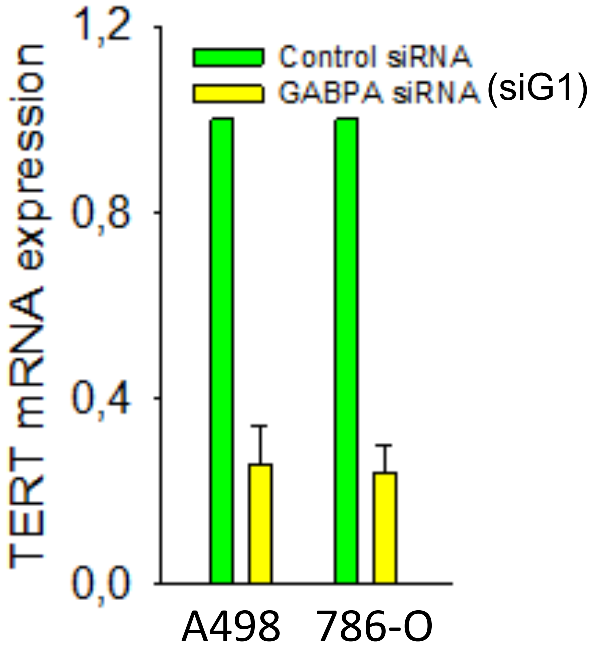


**Figure S2. Downregulation of TERT expression in GABPA-depleted A498 and 786-O cells.** A498 cells carry a wt TERT promoter while 786-O cells have a mutant TERT promoter. Cells were transfected with GABPA siRNA (siG1) and TERT mRNA was assessed using qPCR.
