# Supplementary material for: GABPA-activated TGFBR2 transcription inhibits aggressiveness but is epigenetically erased by oncometabolites in renal cell carcinoma
Source: J Exp Clin Cancer Res. 2022 May 12;41:173. doi: 10.1186/s13046-022-02382-6 (PMC9097325; doi:10.1186/s13046-022-02382-6)
Supplement: Supplementary file 7 — Additional file 7: Figure S3. GABPA overexpression inhibits while itsdepletion promotes the migration of ccRCC-derived cells, respectively. [file 13046_2022_2382_MOESM7_ESM.pdf]

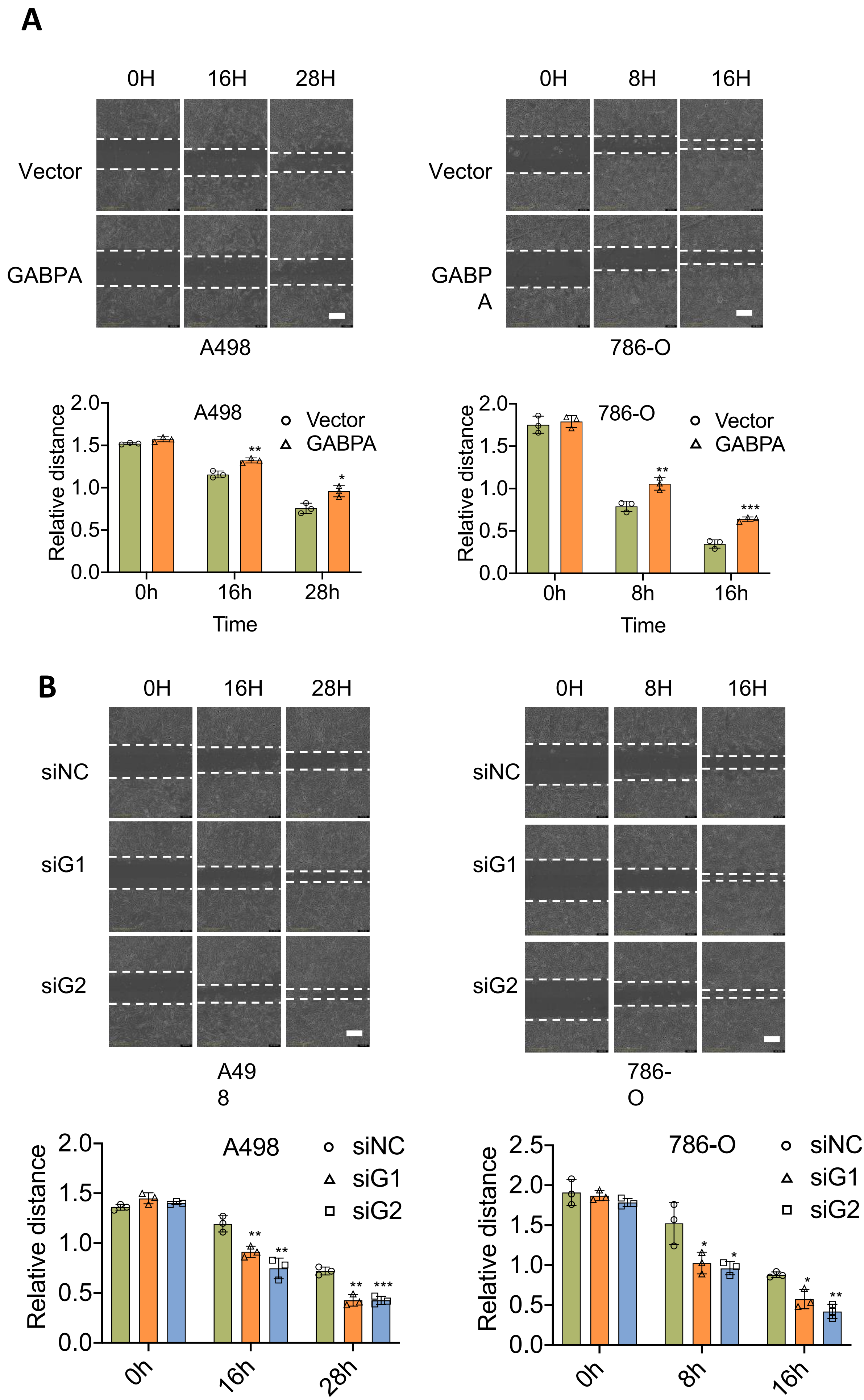

Figure S3. GABPA overexpression inhibits while its depletion promotes the migration of ccRCC-derived cells. (A) A498 and 786-O cells were transfected with control and GABPA expression vectors, respectively, and wound width was assessed at different time points. (B) A498 and 786-O cells were transfected with control and GABPA siRNAs, respectively, and wound width was assessed as above. Scale bars: 100 μM. *, ** and *** denote P<0.05, 0.01 and 0.001, respectively. Three independent experiments were performed.
