# Supplementary material for: GABPA-activated TGFBR2 transcription inhibits aggressiveness but is epigenetically erased by oncometabolites in renal cell carcinoma
Source: J Exp Clin Cancer Res. 2022 May 12;41:173. doi: 10.1186/s13046-022-02382-6 (PMC9097325; doi:10.1186/s13046-022-02382-6)
Supplement: Supplementary file 8 — Additionalfile 8: Figure S4. The correlationbetween GABPA and TGFBR2 mRNA expression ccRCC tumor from GSE73731 andE-MTAB-1980 cohorts. [file 13046_2022_2382_MOESM8_ESM.pdf]

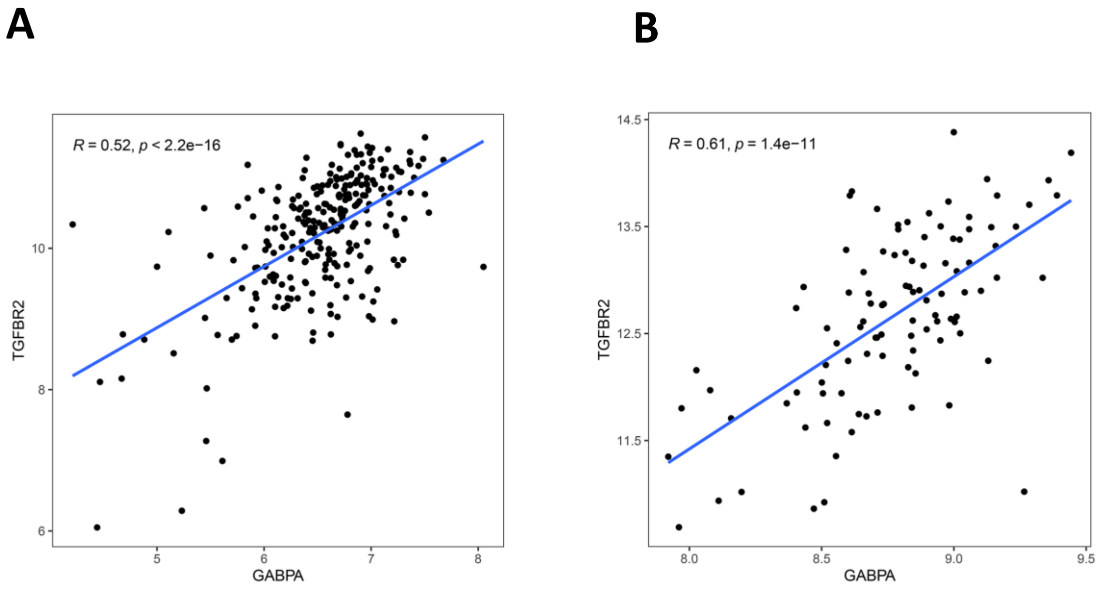

Figure S4. The correlation between GABPA and TGFBR2 mRNA expression in ccRCC tumors from GSE73731 and E-MTAB-1980 cohorts. GSE73731 cohort contains 265 ccRCC tumors analyzed using theAffymetrix Microarrays (A), while RNA sequencing was performed on 101 ccRCC tumors in E-MTAB-1980(B). For RNA seq, mRNA levels were expressed as Log2(RSEM) (RNA-Seq by Expectation-Maximization).mRNA levels assessed using Affymetrix were expressed as log2(probe- set value).
