# Supplementary material for: GABPA-activated TGFBR2 transcription inhibits aggressiveness but is epigenetically erased by oncometabolites in renal cell carcinoma
Source: J Exp Clin Cancer Res. 2022 May 12;41:173. doi: 10.1186/s13046-022-02382-6 (PMC9097325; doi:10.1186/s13046-022-02382-6)
Supplement: Supplementary file 9 — Additionalfile 9: Figure S5. ChIP-seq shows strong GABPA signals on the TGFBR2promoter in Leukemic K562 and liver cancer HepG2 cells. [file 13046_2022_2382_MOESM9_ESM.pdf]

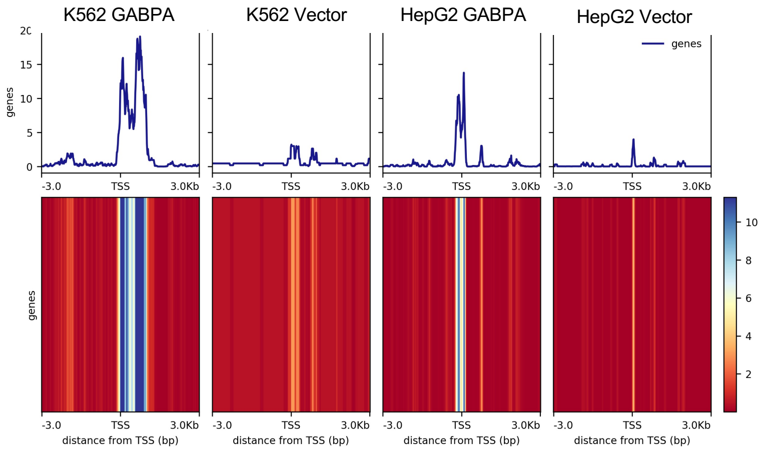

Figure S5. ChIP-seq shows strong GABPA signals on the TGFBR2 promoter in leukemic K562 and liver cancer HepG2 cells. GABPA ChIP-seq results were obtained from GEO dataset (GSM2527661 and GSM2527662) and K562 cells (GSM2825959 and GSM2825960).
