# Supplementary material for: GABPA-activated TGFBR2 transcription inhibits aggressiveness but is epigenetically erased by oncometabolites in renal cell carcinoma
Source: J Exp Clin Cancer Res. 2022 May 12;41:173. doi: 10.1186/s13046-022-02382-6 (PMC9097325; doi:10.1186/s13046-022-02382-6)
Supplement: Supplementary file 10 — Additional file 10: Figure S6. KEGG analyses of RNA seq data from GABPA-depleted 786-O cells show theenrichments of RCC pathways. [file 13046_2022_2382_MOESM10_ESM.pdf]

siGABPA_VS_siNC_kegg_up_down

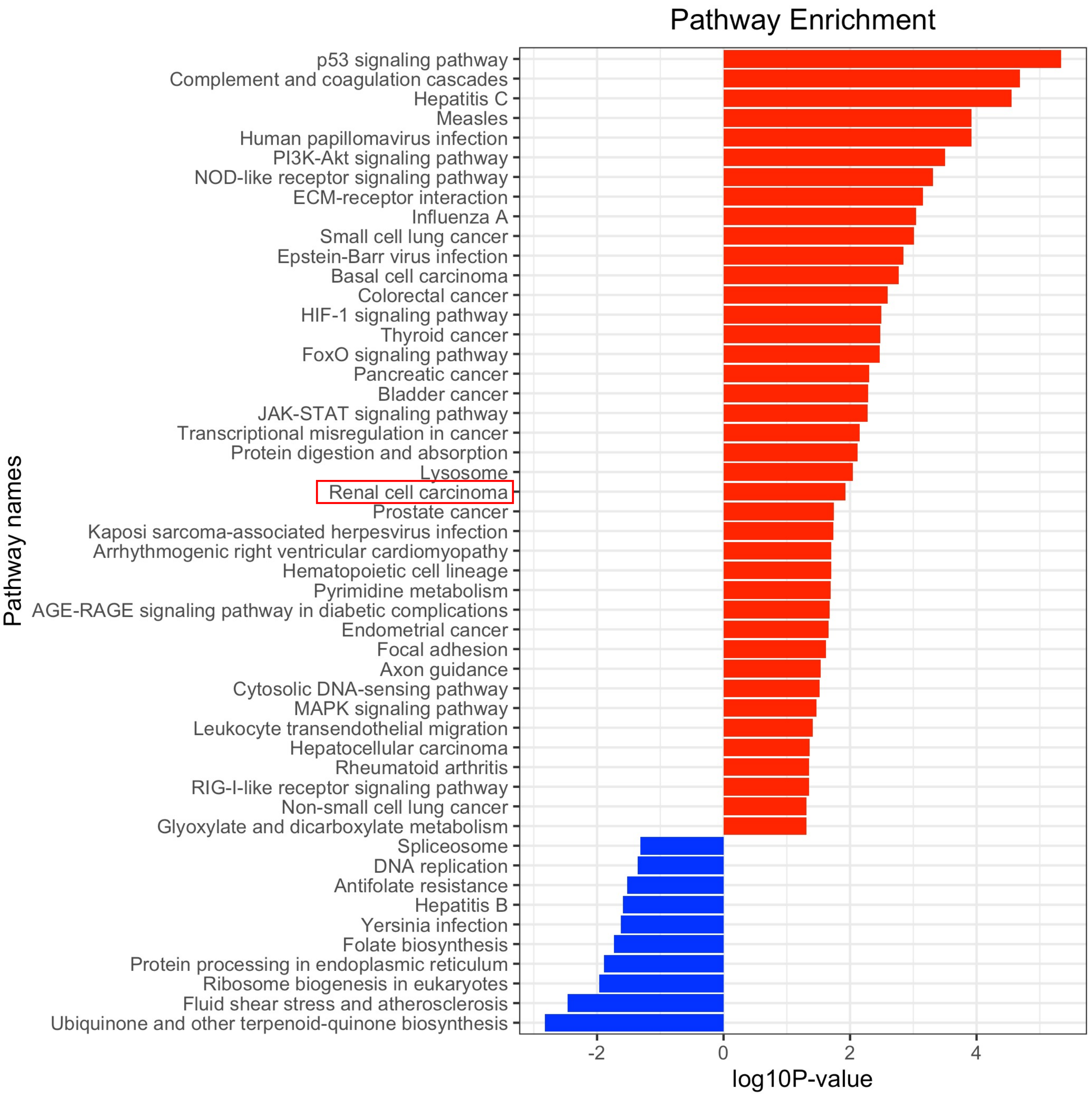


**Figure S6. KEGG analyses of RNA seq data from GABPA-depleted 786-O cells show the enrichments of RCC pathways (Marked in RED)**
