# Supplementary material for: GABPA-activated TGFBR2 transcription inhibits aggressiveness but is epigenetically erased by oncometabolites in renal cell carcinoma
Source: J Exp Clin Cancer Res. 2022 May 12;41:173. doi: 10.1186/s13046-022-02382-6 (PMC9097325; doi:10.1186/s13046-022-02382-6)
Supplement: Supplementary file 11 — Additionalfile 11: Figure S7. L-2-HG promotes while L-2HGDH over-expression or MDH2and LDHB depletion inhibit the proliferation and invasion of ccRCC-derivedcells. [file 13046_2022_2382_MOESM11_ESM.pdf]

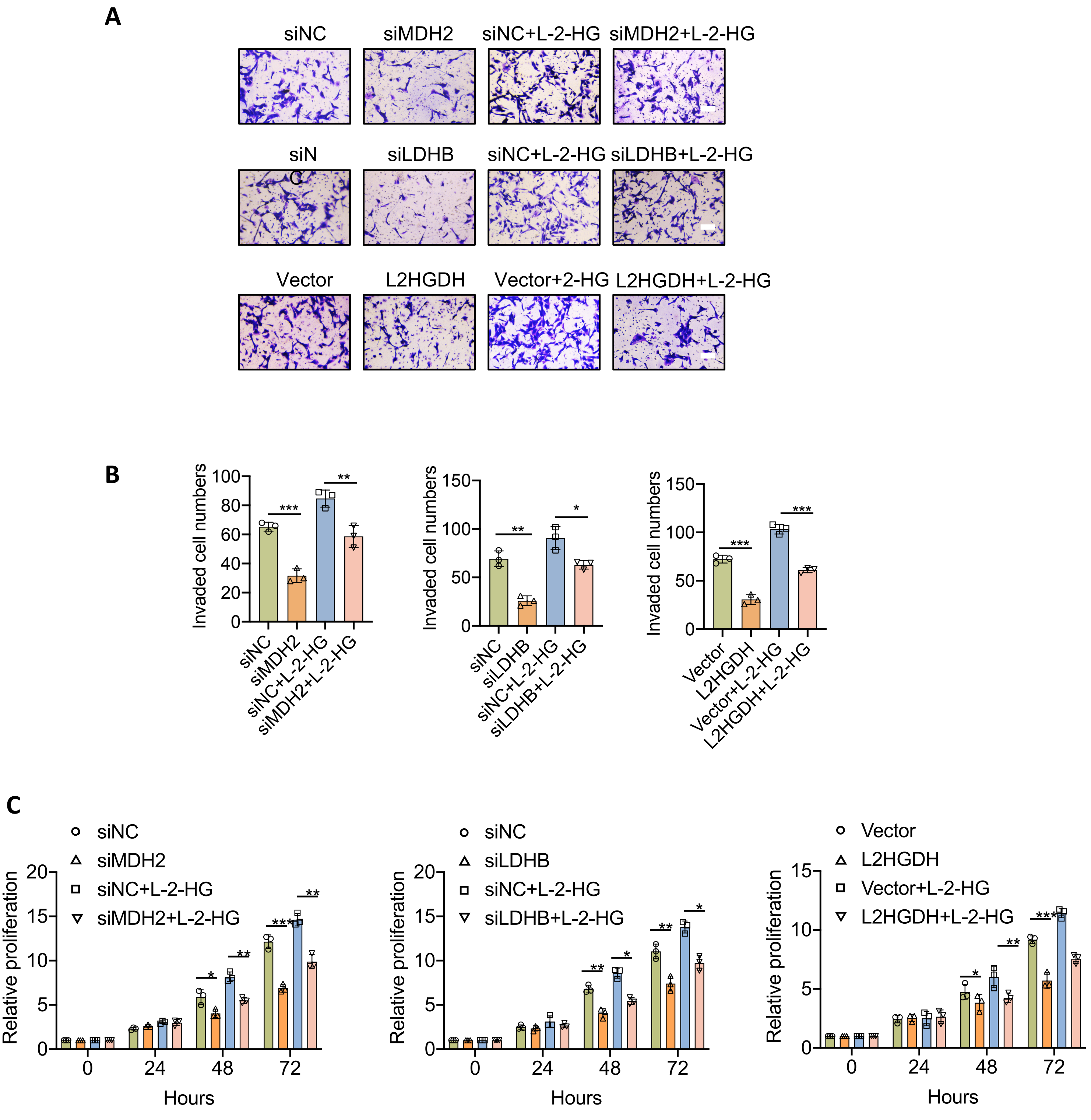

Figure S7. L-2-HG promotes while L-2HGDH over-expression or MDH2 and LDHB depletion inhibit the proliferation and invasion of ccRCC-derived cells. (A) 786-O cells were transfected with MDH2 siRNA(top), LDHB siRNA (Middle) and L2HGDH expression vector (Bottom), respectively, with and without L-2-HG. Invasion was then assessed 48 hours post-transfection using matrigel-coated invasion assays (B). (C) 786-O cells treated above were analyzed for their proliferation using IncuCyte S3 Live-Cell Analysis System. Scale bars: 100 μM. *, ** and *** denote P<0.05, 0.01 and 0.001, respectively. Three independent experiments were performed.
