# Supplementary material for: GABPA-activated TGFBR2 transcription inhibits aggressiveness but is epigenetically erased by oncometabolites in renal cell carcinoma
Source: J Exp Clin Cancer Res. 2022 May 12;41:173. doi: 10.1186/s13046-022-02382-6 (PMC9097325; doi:10.1186/s13046-022-02382-6)
Supplement: Supplementary file 12 — Additional file 12: Figure S8. The lack of correlation between TGFBR2 mRNA expression and its genecopies. [file 13046_2022_2382_MOESM12_ESM.pdf]

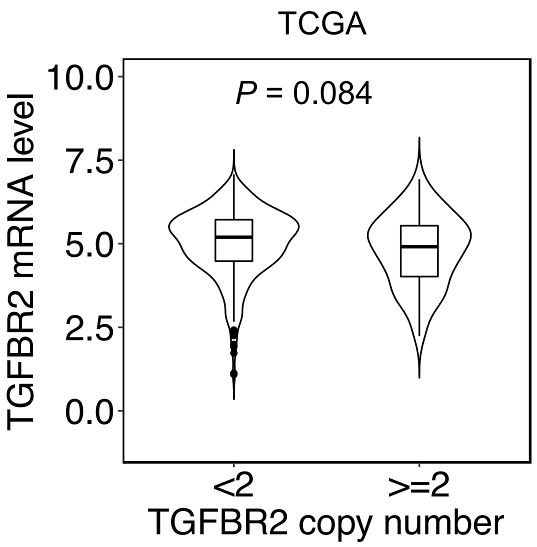

Figure S8. The lack of correlation between TGFBR2 mRNA expression and its gene copies. The TCGA cohort of ccRCC patients were analyzed for TGFBR2 mRNA levels and copy numbers in their tumors . The tumors were divided into two groups with the TGFBR2 copies <2 and >/= 2. TGFBR2 mRNA was expressed as RSEM (RNA-Seq by Expectation Maximization).
